# Production of galactosylated complex-type N-glycans in glycoengineered *Saccharomyces cerevisiae*

**DOI:** 10.1007/s00253-021-11727-8

**Published:** 2021-12-15

**Authors:** Mari A. Piirainen, Heidi Salminen, Alexander D. Frey

**Affiliations:** Department of Bioproducts and Biosystems, Aalto University, Espoo, Finland

**Keywords:** Galactosyltransferase, Glucosamine, Glycoengineering, UDP-glucose 4-epimerase, Yeast

## Abstract

**Abstract:**

N-glycosylation is an important posttranslational modification affecting the properties and quality of therapeutic proteins. Glycoengineering in yeast aims to produce proteins carrying human-compatible glycosylation, enabling the production of therapeutic proteins in yeasts. In this work, we demonstrate further development and characterization of a glycoengineering strategy in a *Saccharomyces cerevisiae* Δ*alg3* Δ*alg11* strain where a truncated Man_3_GlcNAc_2_ glycan precursor is formed due to a disrupted lipid-linked oligosaccharide synthesis pathway. We produced galactosylated complex-type and hybrid-like N-glycans by expressing a human galactosyltransferase fusion protein both with and without a UDP-glucose 4-epimerase domain from *Schizosaccharomyces pombe*. Our results showed that the presence of the UDP-glucose 4-epimerase domain was beneficial for the production of digalactosylated complex-type glycans also when extracellular galactose was supplied, suggesting that the positive impact of the UDP-glucose 4-epimerase domain on the galactosylation process can be linked to other processes than its catalytic activity. Moreover, optimization of the expression of human GlcNAc transferases I and II and supplementation of glucosamine in the growth medium increased the formation of galactosylated complex-type glycans. Additionally, we provide further characterization of the interfering mannosylation taking place in the glycoengineered yeast strain.

**Key points:**

• *Glycoengineered Saccharomyces cerevisiae can form galactosylated N-glycans.*

• *Genetic constructs impact the activities of the expressed glycosyltransferases.*

• *Growth medium supplementation increases formation of target N-glycan structure.*

**Supplementary Information:**

The online version contains supplementary material available at 10.1007/s00253-021-11727-8.

## Introduction

Therapeutic proteins are a fast-growing product segment in the pharmaceutical industry, consisting of products such as antibodies, hormones, and vaccines. The increasing production of therapeutic proteins can provide opportunities to develop alternative production platforms for the predominantly mammalian cell-based production processes. Yeasts have many advantages over mammalian cells in biotechnological processes, including their fast growth in inexpensive growth media, ease of genetic manipulation, and lower risk of contamination. Various therapeutic proteins are currently produced by yeast, mainly by *Saccharomyces cerevisiae* (Walsh 2018).

A significant proportion of therapeutic proteins contain N-glycans. N-glycosylation is a heterogeneous and species-specific posttranslational modification, and the presence of N-glycans as well as their structures can have a significant impact on the properties of a protein. For example, the glycan pattern in antibodies can affect their therapeutic efficacy (Kurogochi et al. 2015; Reusch and Tejada 2015). In addition, darbepoetin alpha, a hyperglycosylated variant of recombinant human erythropoietin, has an increased in vivo activity and serum half-life (Egrie and Browne 2001). The differences in the native N-glycosylation between yeast and mammalian cells currently prevent the use of yeast for the production of therapeutic glycoproteins, as nonhuman N-glycosylation may compromise the therapeutic efficacy and safety of the product.

The N-glycan biosynthesis begins in the endoplasmic reticulum (ER) where in a highly conserved process, a Glc_3_Man_9_GlcNAc_2_ lipid-linked oligosaccharide (LLO) is formed, transferred to a nascent protein by an oligosaccharyltransferase (OST) complex, and trimmed to Man_8_GlcNAc_2_ structure containing three branches during protein folding. Glycans are matured in a species-specific manner as the protein proceeds in the secretory pathway to the Golgi apparatus. The N-glycans in yeast proteins are large and hypermannosylated. In contrast, mammalian glycoproteins mostly contain hybrid and complex-type N-glycans. The maturation of hybrid and complex-type N-glycans begins with the trimming of three α1-2 linked mannose residues, forming Man_5_GlcNAc_2_. Next, a GlcNAc residue is attached via a β1-2 linkage to the α1-3 linked mannose of the A branch by N-acetylglucosaminyltransferase I (GnTI), forming a hybrid-type GlcNAc_1_Man_5_GlcNAc_2_ glycan. Complex-type glycans are formed by further mannosidase trimming followed by the addition of a second GlcNAc residue to the exposed α1-6 linked mannose of GlcNAcMan_3_GlcNAc_2_ by N-acetylglucosaminyltransferase II (GnTII) (Figure S1). The branches of the resulting GlcNAc_2_Man_3_GlcNAc_2_ (G0) or hybrid-type GlcNAc_1_Man_5_GlcNAc_2_ glycans can be further elongated by β1-4-galactosyltransferase (GalT), transferring galactose to the terminal GlcNAc residues. Further on, the galactosylated branches can be capped with sialic acids. In addition to these modifications, additional branching of the glycan core as well as core fucosylation can occur.

During the past two decades, the N-glycosylation pathways of various yeasts have been engineered aiming to enable the production of therapeutic proteins with human-compatible N-glycosylation. An essential step in yeast glycoengineering is to create suitable intermediate N-glycan structures that can act as substrates for the subsequent mammalian-type glycan maturation steps. Some glycoengineering approaches have relied on replicating the glycan trimming reactions of the mammalian glycosylation pathway by expressing mannosidases and blocking the formation of the yeast-specific outer chain by *OCH1* deletion (Choi et al. 2003; Hamilton et al. 2003; Vervecken et al. 2004). An alternative approach is to either partially or completely bypass the glycan trimming steps. This can be achieved by deleting mannosyltransferases in the LLO biosynthesis pathway, preventing the elongation of the LLO branches (Bobrowicz et al. 2004; Cheon et al. 2012; De Pourcq et al. 2012; Wang et al. 2013). In *S. cerevisiae*, deletion of *ALG3* and *ALG11* genes (Parsaie Nasab et al. 2013) resulted in a strain that forms a truncated trimannosyl core structure (Man_3_GlcNAc_2_) in the ER. This glycan can be directly converted to complex-type glycans without any trimming reactions.

After a suitable precursor glycan is formed, hybrid and complex-type glycans are generated in yeasts by expressing mammalian N-acetylglucosaminyltransferases, galactosyltransferases, and sialyltransferases. Glycosyltransferases in the Golgi apparatus are type II transmembrane proteins, and their N-terminal cytoplasmic, transmembrane, and stem domains direct the transferases into certain Golgi cisternae (Tu and Banfield 2010). When expressing glycosyltransferases of mammalian origin in yeast, their correct localization is ensured by fusing their catalytic domains to the localization sequences of yeast endogenous glycosyltransferases. Mammalian glycosyltransferases also utilize nucleotide sugars including UDP-GlcNAc and UDP-galactose not present in the Golgi apparatus of most yeasts and improving their availability has often been required to obtain efficient conversions by the mammalian glycosyltransferases. Expression of a UDP-GlcNAc transporter from *Kluyveromyces lactis* (Yea4, alternatively called Mnn2-2) has increased the amount of hybrid and complex-type glycans in *S. cerevisiae* and other yeasts (Choi et al. 2003; Bobrowicz et al. 2004; Wang et al. 2013; Piirainen et al. 2016). In addition, incorporation of a UDP-glucose 4-epimerase from *Schizosaccharomyces pombe* into a GalT fusion protein has provided efficient N-glycan galactosylation (Bobrowicz et al. 2004; Jacobs et al. 2009; Wang et al. 2013). With these and additional modifications, yeast strains forming hybrid and complex-type glycans with terminal GlcNAc (Callewaert et al. 2001; Choi et al. 2003; Hamilton et al. 2003; Cheon et al. 2012; Parsaie Nasab et al. 2013), galactose (Vervecken et al. 2004; Bobrowicz et al. 2004; Wang et al. 2013), and even sialic acid residues (Hamilton et al. 2006) have been developed.

We have previously reported that complex-type glycans bearing terminal GlcNAc residues can be generated in *S. cerevisiae* and that their amount can be increased by enhancing the transport of UDP-GlcNAc to the Golgi apparatus (Parsaie Nasab et al. 2013; Piirainen et al. 2016). In this work, we build on this glycoengineering approach and demonstrate galactosylation of hybrid-like and complex-type N-glycans by the expression of a galactosyltransferase fusion protein. In addition, we show that the formation of these glycans can be enhanced by optimization of expression vectors and cultivation medium.

## Materials and methods

### Strains

*S. cerevisiae* host strain and plasmids used in this study are presented in Table 1. Strain YMP14 (Piirainen et al. 2016) is derived from the glycoengineered strain YAF39 that contains deletions of *ALG3* and *ALG11* genes, the artificial flippase Flc2*, and an oligosaccharyltransferase from *Leishmania brasiliensis*, and has been UV-mutagenized for improved growth (Parsaie Nasab et al. 2013). YMP17 was generated from strain YMP14 by removing the nourseothricin selection marker from *MNN1* locus with Cre recombinase (Hegemann and Heick 2011).

**Table 1:** Strains and plasmids used in this work

Name	Genotype/description	Reference
SS328	*MATα ade2-101 his3Δ200 lys2-801 ura3-52*	ATCC® MYA193™
YMP17	SS328 ∆*alg3*::*HIS3* ∆*alg11*::*HIS3* ∆*leu2*::*KanMX4*::LbSTT3_3::Flc2* *Δmnn1*::loxP, UV-mut	This work
pEK7	Empty low copy plasmid, *LEU2* selection marker	de Ruijter et al. (2016)
pAK3	ORF for Kre2p-GnTI fusion under control of *GAL1* promoter, *URA3* selection marker, low copy plasmid	This work
pAX428	ORFs for Kre2p-GnTI and Mnn2p-GnTII fusions under control of *GAL1-10* promoter, *URA3* selection marker, high copy plasmid	Parsaie Nasab et al. (2013)
pSKH01	ORFs for Kre2p-GnTI and Mnn2p-GnTII fusions under control of *GAL1* promoters, *URA3* selection marker, low copy plasmid	This work
pAF21	ORFs for Kre2p-GnTI and Mnn2p-GnTII fusions under control of *GAL1* promoters, KlYea4p under control of *GPD* promoter, *URA3* selection marker, low copy plasmid	This work
pAF22	ORFs for Kre2p-GnTI fusion under control of *GAL1* promoter, KlYea4p under control of *GPD* promoter, *URA3* selection marker, low copy plasmid	This work
pSR01	ORF for Mnn2p-Uge1p-GalT fusion under control of *GAL1* promoter, *LEU2* selection marker, low copy plasmid	This work
pSR02	ORF for Mnn2p-GalT fusion under control of *GAL1* promoter, *LEU2* selection marker, low copy plasmid	This work

### Cloning of overexpression constructs

All recombinant DNA work was done using *Escherichia coli* TOP10 (Invitrogen, Waltham, MA, USA) as the cloning host. Empty plasmid pEK7 containing a *LEU2* selection marker was used to complement for auxotrophies in strains where no gene expression with a *LEU2* plasmid took place (de Ruijter et al. 2016). Expression vectors are based on pRS plasmid series (Mumberg et al. 1995), and oligonucleotides used for generating the plasmids are listed in Table S1.

Human GlcNAc transferase I and II fusion proteins with yeast endogenous localization sequences have been constructed by Parsaie Nasab et al. (2013). GlcNAc transferase I fusion protein (GnTI) consists of *S. cerevisiae* Kre2p targeting sequence, encompassing the N-terminal cytoplasmic, transmembrane and stem domains, the catalytic domain of human GlcNAc transferase I, and a C-terminal FLAG tag, and GlcNAc transferase II fusion protein (GnTII) consists of *S. cerevisiae* Mnn2p targeting sequence, the catalytic domain of human GlcNAc transferase II, and a C-terminal FLAG tag. A low copy number expression vector for GnTI was constructed by inserting the ORF encoding Kre2p-GnTI-FLAG into *Spe*I and *Xho*I sites of pRS416-GAL by standard cloning procedures, creating plasmid pAK3. A low copy number vector for coexpression of GnTI and GnTII under the control of *GAL1* promoter (GnTI/II vector) was constructed in two steps. First, ORF encoding Mnn2p-GnTII-FLAG was amplified from pAX428 (Parsaie Nasab et al. 2013) by PCR with oligonucleotides OMP97 and OMP98 and inserted into *Spe*I and *Xho*I site of pRS416-GAL with ELIC cloning (Koskela and Frey 2014), creating plasmid pMP27. Next, a DNA fragment containing *GAL1* promoter, ORF encoding Kre2p-GnTI-FLAG, and *CYC1* terminator was amplified from pAK3 by PCR with oligonucleotides OMP108 and OMP109 and inserted into *Eco*53kI site of pMP27 with ELIC cloning, creating plasmid pSKH01.

The expression cassette for UDP-GlcNAc transporter from *K. lactis* (Yea4) was combined with GnTI and GnTI/II expression vectors as follows. First, a fragment containing *GPD1* promoter and *KlYEA4* ORF was excised from pMP002 (Piirainen et al. 2016) with *Sac*I and *Xho*I and inserted into *Sac*I and *Xho*I site of pMP27, creating plasmid pAF19 where the *KlYEA4* expression construct is in an expression vector containing *URA3* selection marker. Next, a DNA fragment containing *GAL1* promoter, ORF encoding Mnn2p-GnTII-FLAG, and *CYC1* terminator was excised from pMP27 with *Eco*53kI and *Sbf*I and inserted into *Nae*I and *Sbf*I site of pAF19, creating plasmid pAF20. A DNA fragment containing *GAL1* promoter, ORF encoding Kre2p-GnTI-FLAG, and *CYC1* terminator was then amplified from pAK3 with oligonucleotides OMP104 and OMP105 and inserted into *Nae*I site of pAF19 and pAF20, creating plasmids pAF22 and pAF21, respectively.

For expression of human β1-4 galactosyltransferase 1 (GalT, Uniprot ID P15291) and UDP-glucose 4-epimerase from *S. pombe* (Uge1, Uniprot ID Q9Y7X5), a codon-optimized ORF encoding a fusion protein (Uge1-GalT) containing Uge1, GSGG linker peptide, and amino acids 44–398 of GalT was synthesized (Eurofins Genomics) and the sequence is deposited (GenBank accession number OK129337). To generate expression constructs for GalT both with and without Uge1, ORFs encoding Uge1-GalT or GalT were amplified by PCR with oligonucleotide pairs OMP88 and OMP90, or OMP89 and OMP90, respectively. The PCR fragments were inserted by ELIC cloning into *Xho*I and *Bmg*BI-digested pAK2, consisting of pRS415-GAL plasmid backbone and ORF encoding amino acids 1–36 of *S. cerevisiae* Mnn2p localization sequence. The resulting plasmids pSR01 and pSR02 contain the ORFs encoding Mnn2p-Uge1-GalT and Mnn2p-GalT fusion proteins, respectively, under the control of *GAL1* promoter and *CYC1* terminator.

### Cultivation of yeast strains

*S. cerevisiae* strains were cultivated at 28 °C in chemically defined medium (0.67% yeast nitrogen base without amino acids and Hopkins dropout mixture lacking leucine and uracil) supplemented with 0.2 M sorbitol and with 2% raffinose as a carbon source. For N-glycan analysis of cell wall and secreted proteins, 3 ml precultures were grown at 230 rpm for 48–72 h. Cells were diluted to an OD_600_ of 0.2 in 20 ml culture volume and grown until OD_600_ of at least 1 was reached. Cells were harvested, resuspended to an OD_600_ of 1 in 20 ml of fresh medium supplemented with 2% galactose for induction of GnTI, GnTII, and GalT expression, and grown for 24 h. Induction medium for growth medium supplementation tests and for N-glycan analysis of secreted proteins was supplemented with 15 mM glucosamine.

### N-glycan sample preparation

N-glycans were isolated from the cell wall proteins and secreted proteins. Cell wall proteins were isolated and prepared for N-glycan analysis as previously described (Piirainen et al. 2019). Briefly, 50 OD_600_ units of the cells were lysed using 0.5 mm glass beads in 10 mM Tris–HCl buffer pH 7.4 containing protease inhibitor cocktail (Complete EDTA-free, Roche, Basel, Switzerland) and 1 mM phenylmethanesulfonyl fluoride. Covalently linked cell wall material was collected by centrifugation (16,000 g, 1 min), and the reduction and alkylation of cysteines were performed at 37 °C in 10 mM dithiotreitol in denaturing buffer (2 M thiourea, 7 M urea, 2% sodium dodecyl sulphate, 50 mM Tris–HCl pH 8) followed by addition of iodoacetamide to a final concentration of 24 mM. The pellet was washed five times with the denaturing buffer and five times with water. For the analysis of the N-glycans on secreted proteins, 15 ml of culture medium was cleared by centrifugation, and the supernatant was concentrated and washed with water to a volume of 200–300 µl using centrifugal concentrators with a molecular weight cut-off of 10,000 (Sartorius, Göttingen, Germany).

N-glycans of both cell wall and secreted proteins were released in 200 µl of reaction mixture containing 1 µl peptide-N-glycosidase F (500 U/µl PNGase F, glycerol free, New England Biolabs, Ipswich, MA, USA), glycoprotein denaturing buffer, GlycoBuffer 2, and 1% NP-40 at 37 °C with shaking at 230 rpm for 16 h. Secreted protein samples were denatured in glycoprotein denaturing buffer (New England Biolabs, Ipswich, MA, USA) for 10 min at 95 °C prior to the PNGase F reaction. N-glycans were purified with C18 and graphitized carbon columns (Supelclean ENVI-18 and ENVI-carb, Sigma Aldrich, St. Louis, MO, USA) as described in Piirainen et al. (2019). For MS/MS glycan analysis, N-glycan samples were permethylated as described earlier (Piirainen et al. 2019), except that chloroform was replaced by dichloromethane.

### Glycosidase reactions

α1-2-mannosidase (Agilent, Santa Clara, CA, USA) and β1-4-galactosidase (New England Biolabs, Ipswich, MA, USA) reactions were performed according to manufacturer’s instructions, and N-glycans were purified from the reaction mixture with HyperSep™ Hypercarb microscale solid phase extraction tips (Sigma Aldrich, St. Louis, MO, USA). The tips were washed 5 times with 20 µl of 95% acetonitrile and equilibrated 5 times with 20 µl of 2% acetonitrile. Samples were loaded to the tips by pipetting 20–50 times, the tips were washed 10 times with 20 µl of 2% acetonitrile, and glycans were eluted by pipetting 10 times with 10 µl of 70% acetonitrile, repeating elution by pipetting 3 times, and combining the eluate with the first eluate. The samples were dried with air flow at 60 °C.

#### MALDI-TOF

N-glycan samples were dissolved in 10 µl of water prior to MS analysis. Equal amounts of sample and matrix solution (20 mg/ml of super-DHB [9:1 mixture of 2,5-dihydroxybenzoic acid and 2-hydroxy-5-methoxybenzoic acid] in 30% acetonitrile, 0.1% trifluoroacetic acid, and 1 mM NaCl) were mixed and spotted onto a target plate. N-glycans and LLOs were analyzed by UltrafleXtreme MALDI-TOF MS operated in positive ion and reflector mode (Bruker Daltonics, Billerica, MA, USA). Permethylated N-glycan samples were dissolved in 5% acetonitrile containing 0.1% trifluoroacetic acid and spotted onto a target plate with the super-DHB matrix. MS/MS spectra were acquired in positive LIFT mode with high-energy CID using argon as a collision gas. For MS/MS measurements in negative ion mode, samples were applied as described earlier (Domann et al. 2012) with minor modifications. In short, 0.5 µl of N-glycan sample was spotted onto a target plate followed by 0.3 µl of THAP matrix (150 mg/ml 2′,4′,6′-trihydroxyacetophenone monohydrate in acetone) and 0.5 µl of 1 M ammonium nitrate. MS/MS spectra were acquired in negative LIFT mode with CID using argon as a collision gas. Annotation of glycans in figures follows the symbol nomenclature for glycans (Varki et al. 2015), depicting hexoses as filled circles (glucose: blue; mannose: green; galactose: yellow) and N-acetylhexoses as filled squares (N-acetylglucosamine: blue).

### Data analysis

The efficiencies of the GnTI, GnTII, and GalT reactions were calculated based on the relative glycan abundances obtained from MALDI-TOF MS measurements of nontreated N-glycan samples and N-glycan samples sequentially digested with α1-2 mannosidase and β1-4 galactosidase. The enzymatic efficiencies were defined as the total relative abundance of glycan structures processed by GnTI, GnTII, or GalT per the total relative abundance of potential substrates available for the respective enzymes. All glycans containing an α1-3 linked mannose residue (Man_3-6_GlcNAc_2_, Gal_0-1_GlcNAc_1_Man_3-4_GlcNAc_2_, Gal_0-2_GlcNAc_2_Man_3_GlcNAc_2_) were considered as substrates for GnTI, all glycans processed by GnTI were considered as substrates for GnTII, and all β1-2 linked GlcNAc residues were considered as substrates for GalT. Statistical significance of the differences in the relative glycan abundances between strains was evaluated by Student’s *t* test.

## Results

### Optimization of the GlcNAc_2_Man_3_GlcNAc_2_ synthesis pathway

We have previously shown that complex-type GlcNAc_2_Man_3_GlcNAc_2_ (G0) glycans can be formed in a glycoengineered Δ*alg3* Δ*alg11 S. cerevisiae* strain and that its amount can be increased by the expression of a UDP-GlcNAc transporter from *K. lactis* (Yea4) (Parsaie Nasab et al. 2013; Piirainen et al. 2016). When aiming to develop the strain further to obtain galactosylated N-glycans, the maximal achievable level of galactosylation depends on the amount of substrate glycans available for GalT. It is therefore important to optimize the glycan modification steps preceding galactosylation. We systematically tested a number of existing and new plasmids for the expression of GnTI, GnTII, and Yea4 in strain YMP17, which is a derivative of a glycoengineered strain YMP14 producing less interfering mannosylation due to *MNN1* deletion (Piirainen et al. 2016). The N-glycan pattern of cell wall proteins in these strains was analyzed by MALDI-TOF MS and relative abundances of the N-glycan structures were calculated (Table S2). To evaluate the different genetic constructs, we also estimated the efficiencies of each glycan-modifying reaction based on the MALDI-TOF MS data by calculating the amount of glycans processed by each glycosyltransferase relative to the amount of their theoretically available substrate sites (Table 2).

**Table 2:** Efficiencies of GnTI, GnTII, and GalT in cell wall N-glycan samples

Expressed enzymes	GnTI efficiency^a^ (%)	GnTII efficiency^a^ (%)	GalT efficiency^b^ (%)
GnTI	53.1 ± 0.8	n/a	n/a
GnTI + Yea4	50.3 ± 3.8	n/a	n/a
GnTI + Yea4 + GalT	51.3 ± 2.2	n/a	77.5
GnTI + Yea4 + Uge1-GalT	57.0 ± 1.1	n/a	83.2
GnTI + II (high copy plasmid)	32.6 ± 3.2	70.4 ± 6.4	n/a
GnTI + II	46.5 ± 3.9	77.9 ± 4.0	n/a
GnTI + II + Yea4	50.7 ± 2.7	92.2 ± 0.4	n/a
GnTI + II + Yea4 with glucosamine	50.5	92.2	n/a
GnTI + II + Yea4 + GalT	37.7 ± 2.5	50.6 ± 2.1	58.6
GnTI + II + Yea4 + Uge1-GalT	41.8 ± 2.7	62.9 ± 8.4	60.3
GnTI + II + Yea4 + Uge1-GalT with glucosamine	53.3 ± 0.2	87.6 ± 0.8	64.1

^a^The data represent the mean ± SEM of 3–5 biological replicates, except for the strain expressing GnTI + II + Yea4 with glucosamine the mean of 2 biological replicates is shown. ^b^GalT efficiency was calculated from galactosidase digestions performed to a representative sample

*n/a*, not applicable

**Fig. 1:**
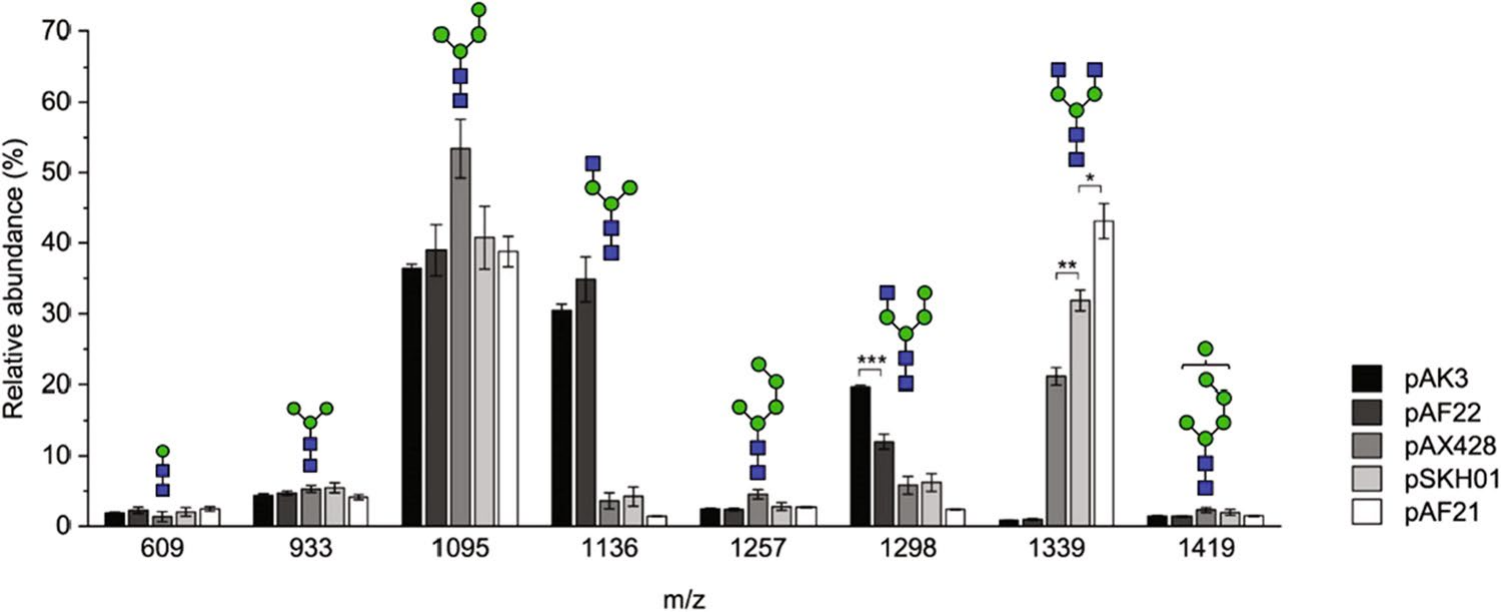
Relative abundances of N-glycans isolated from the cell walls of YMP17 expressing GnTI (pAK3), GnTI and *KlYEA4* (pAF22), GnTI and GnTII under *GAL1-10* promoter in a high copy number plasmid (pAX428), GnTI and GnTII in a low copy number plasmid (pSKH01), and GnTI, GnTII, and *KlYEA4* in a low copy number plasmid (pAF21) measured by MALDI-TOF MS. The data represent the mean ± SEM of 3–5 biological replicates. *p* < 0.05 (*), *p* < 0.01 (**), *p* < 0.001 (***)

**Fig. 2:**
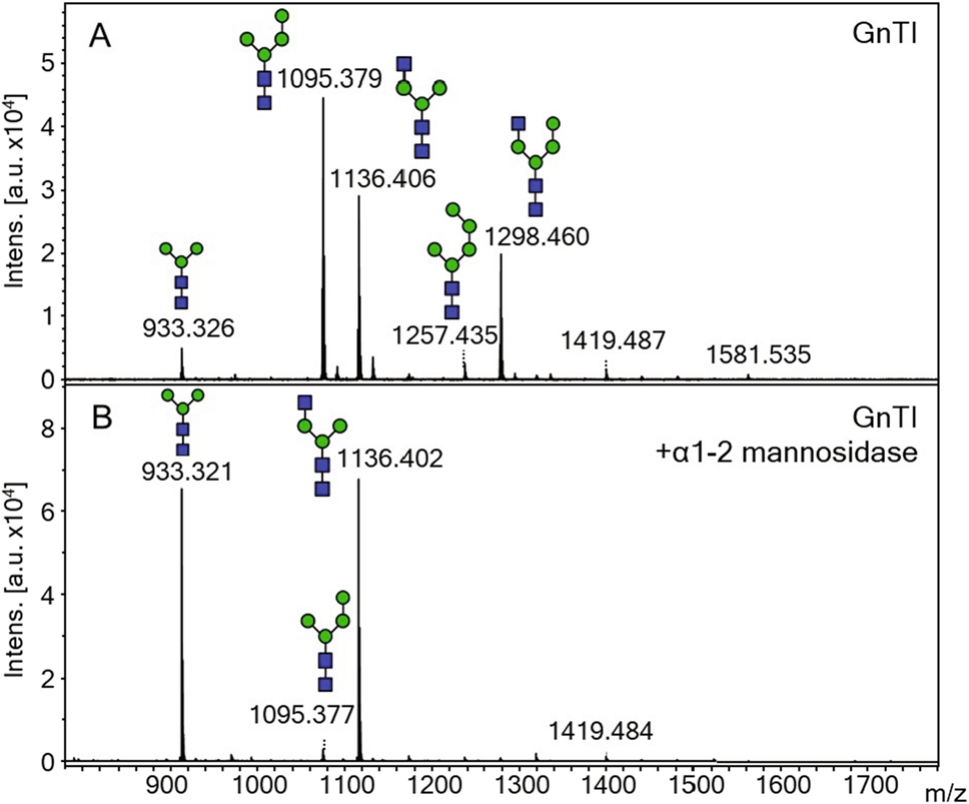
MALDI-TOF MS spectra of N-glycans isolated from cell wall proteins of YMP17 expressing GnTI in plasmid pAK3 before (**A**) and after (**B**) α1-2 mannosidase treatment

When GnTI was expressed in strain YMP17, approximately half of total cell wall N-glycans received a GlcNAc residue, and 39% of these glycans additionally contained a fourth mannose residue (Table S2). These GlcNAcMan_3-4_GlcNAc_2_ structures resemble mammalian hybrid-type glycans except that due to the *ALG3* deletion and endogenous mannosyltransferase activity of *S. cerevisiae*, the α1-6 linked arm of the trimannosyl core contains no mannose extensions or a single additional mannose whereas the α1-6 linked arm of hybrid-type glycans contains one α1-3 linked and one α1-6 linked mannose residue. The glycans not processed by GnTI predominantly consisted of Man_4_GlcNAc_2_ structures that had received one additional mannose residue to the trimannosyl core (Fig. 1). The fourth mannose residue in both Man_4_GlcNAc_2_ and GlcNAcMan_4_GlcNAc_2_ structures was confirmed to be α1-2 linked (Fig. 2), which is in accordance with earlier findings of the interfering mannose (Parsaie Nasab et al. 2013).

Next, we coexpressed Yea4 with GnTI to test whether the positive effects of Yea4 on glycan pattern reported earlier are related to the activity of GnTI. When Yea4 was coexpressed with GnTI, the relative abundance of glycans processed by GnTI did not change significantly (Table 2). However, the relative abundance of GlcNAcMan_4_GlcNAc_2_ decreased (Fig. 1, Table S2), only composing 26% of all glycans processed by GnTI. Thus, expression of a UDP-GlcNAc transporter did not increase the GnTI activity but decreased mannosylation of the GlcNAcMan_3_GlcNAc_2_ structure. The decreased mannosylation was only observed in glycans processed by GnTI, as the relative amounts of Man_3_GlcNAc_2_ and Man_4_GlcNAc_2_ remained unchanged.

In our earlier study, GnTI and GnTII were expressed in a high copy number vector using a bidirectional *GAL1-10* promoter, placing GnTI and GnTII under *GAL1* and *GAL10* promoters, respectively (Parsaie Nasab et al. 2013). When this vector was expressed in YMP17, the relative abundance of the G0 glycan was 21% (Fig. 1, Table S3). The efficiency of the GnTI reaction in this strain was only 33% (Table 2), significantly lower than obtained with the low copy number GnTI expression vector. We therefore wondered if changing the expression construct for GnTI and GnTII to a low copy number version could improve the yield of complex-type glycans, and created a low copy number vector containing both GnTI and GnTII under *GAL1* promoter. With the new GnTI/II expression vector, the relative abundance of the G0 glycan increased to 32% (Fig. 1, Table S3). This improvement was partially caused by an increased GnTI efficiency, which was nearly as high as when expressed without GnTII. The relative abundance of Man_4_GlcNAc_2_ decreased correspondingly, although biological variation was high. In addition, the amount of glycans processed by GnTII increased. Taking into account that GnTII only accepts glycans processed by GnTI as its substrate (Vella et al. 1984), the efficiency of the GnTII reaction was 70% with the high copy number version and 78% with the new low copy GnTI/II expression vector (Table 2).

After demonstrating that the new version of the GnTI/II expression vector provided increased relative abundances of G0 glycans, we incorporated the expression of the UDP-GlcNAc transporter to this vector to further increase its amounts. With Yea4 coexpression, the relative abundance of G0 increased to 43% (Fig. 1, Table S3), exceeding the levels of the previously predominant Man_4_GlcNAc_2_ structure. Yea4 expression did not significantly increase the efficiency of GnTI, similarly to our observations without GnTII expression. However, GnTII efficiency of over 90% was obtained when Yea4 was expressed (Table 2). In addition, very low amounts of incompletely processed or interfering structures other than Man_4_GlcNAc_2_ were seen. Thus, the coexpression of Yea4 with GnTI and GnTII led to a very high GnTII activity in our glycoengineered system, but the activity of GnTI was modest and mostly unaffected by the expression of Yea4 or GnTII.

### Galactosylation of hybrid-like and complex glycans in *S. cerevisiae*

**Fig. 3:**
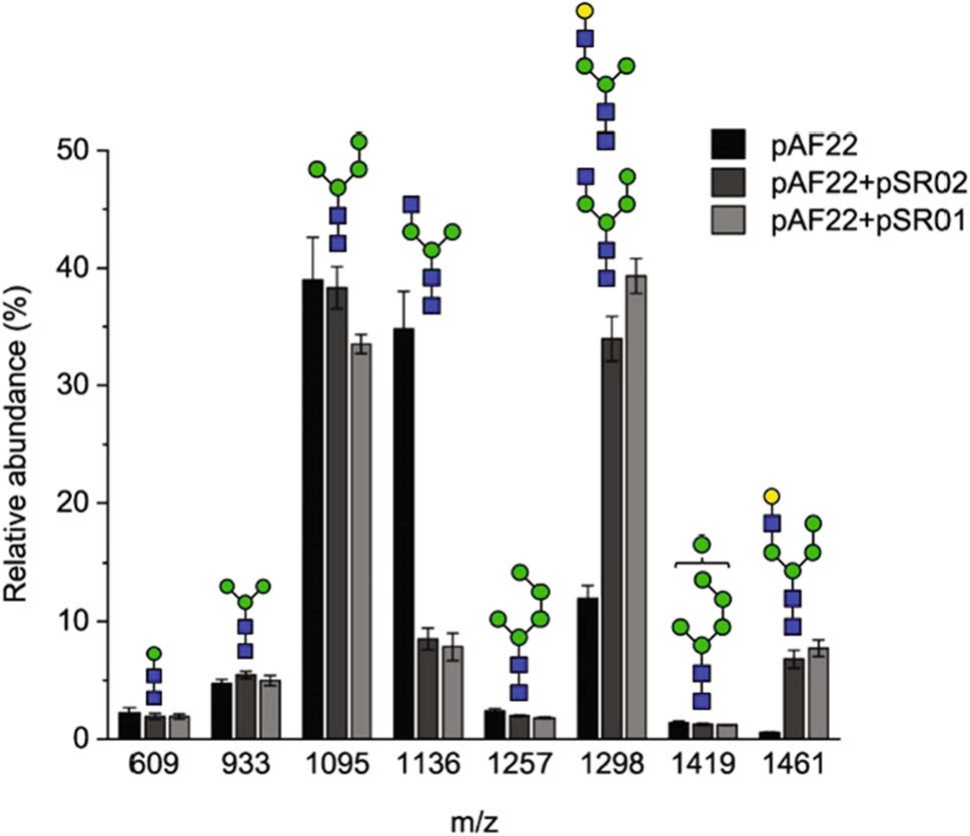
Relative abundances of N-glycans isolated from the cell walls of YMP17 expressing GnTI and *KlYEA4* (pAF22), GnTI, *KlYEA4* and GalT (pAF22 + pSR02), and GnTI, *KlYEA4*, and Uge1-GalT (pAF22 + pSR01) measured by MALDI-TOF MS. The data represent the mean ± SEM of three biological replicates

Next, we aimed to test whether the complex and hybrid-like glycans formed in *S. cerevisiae* could be galactosylated. We constructed a tripartite fusion protein (Uge1-GalT) consisting of Mnn2p targeting sequence, UDP-glucose 4-epimerase from *S. pombe*, a GSGG linker peptide, and GalT that was used successfully for galactosylation of N-glycans in *Pichia pastoris* and *Hansenula polymorpha* (Bobrowicz et al. 2004; Wang et al. 2013). To determine the importance of the epimerase domain in our expression system, we also created a corresponding fusion protein lacking the UDP-glucose 4-epimerase domain (GalT). We first confirmed by Western blot that both Uge1-GalT and GalT fusion proteins were expressed intracellularly and appeared intact (Figure S2). Next, we coexpressed the GalT and Uge1-GalT constructs with GnTI and Yea4 and analyzed the cell wall N-glycans with MALDI-TOF MS. When GalT or Uge1-GalT was expressed, the relative abundances of the signals at m/z 1298 and 1460 increased, corresponding to the sodium adducts of GlcNAcMan_4_GlcNAc_2_ or GalGlcNAcMan_3_GlcNAc_2_, and GlcNAcMan_5_GlcNAc_2_ or GalGlcNAcMan_4_GlcNAc_2_, respectively. Correspondingly, the relative abundance of GlcNAcMan_3_GlcNAc_2_ structure dropped from 35 to under 10% upon expression of Uge1-GalT and GalT (Fig. 3 and Fig. 5, Table S2). Enzymatic digestions of the N-glycans with α1-2 mannosidase and β1-4 galactosidase revealed that nearly 90% of the signal intensity at m/z 1298 and 1460 arose from galactosylated structures, and approximately 80% of all potential GalT substrate sites, i.e., β1-2 linked GlcNAc residues, had received a β1-4 linked galactose residue (Table 2). The presence of the UDP-glucose 4-epimerase domain had at most a minor positive effect on the extent of galactosylation. In addition, the efficiency of the GnTI reaction was not hampered by the expression of GalT or Uge1-GalT. In fact, the GnTI efficiency was even slightly increased when coexpressed with Uge1-GalT. Thus, the expression of GalT or Uge1-GalT does not interfere with GnTI activity and glycans processed with GnTI can be efficiently galactosylated in glycoengineered *S. cerevisiae* strain YMP17 both with and without the UDP-glucose 4-epimerase.

**Fig. 4:**
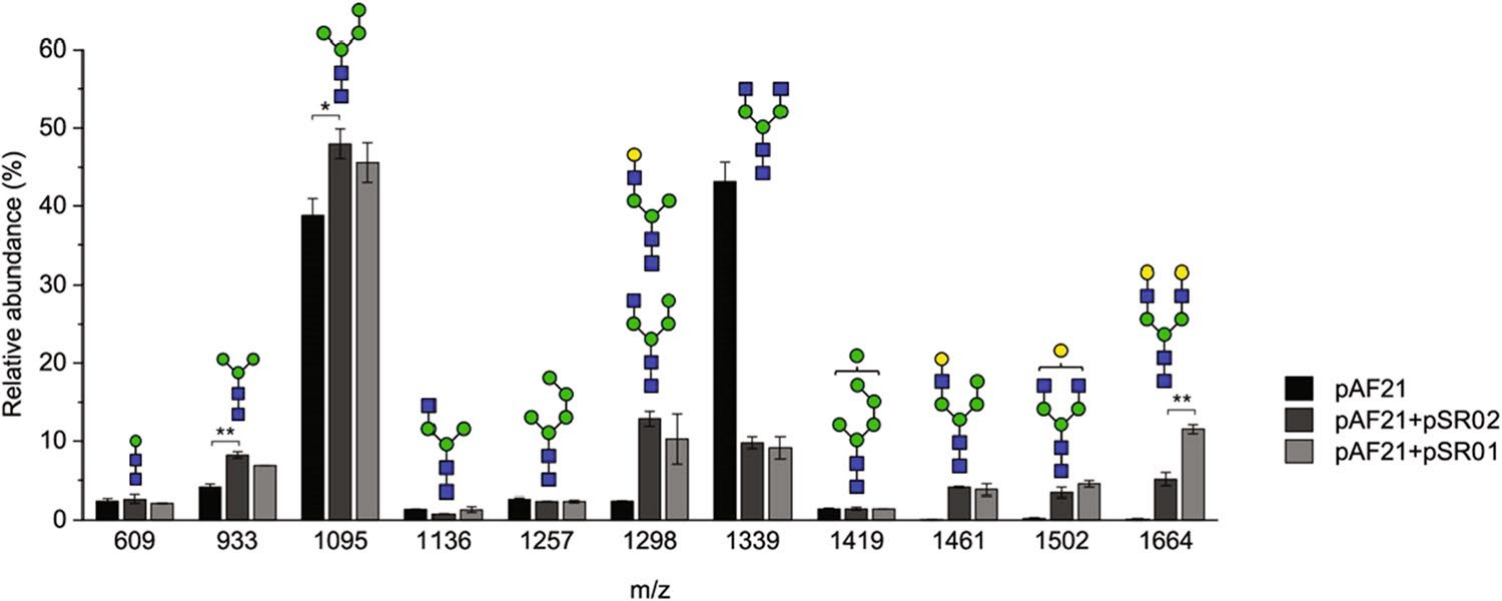
Relative abundances of N-glycans isolated from the cell walls of YMP17 expressing GnTI, GnTII, and *KlYEA4* (pAF21), GnTI, GnTII, *KlYEA4*, and GalT (pAF21 + pSR02), and GnTI, GnTII, *KlYEA4*, and Uge1-GalT (pAF21 + pSR01) measured by MALDI-TOF MS. The data represent the mean ± SEM of three biological replicates. *p* < 0.05 (*), *p* < 0.01 (**)

**Fig. 5:**
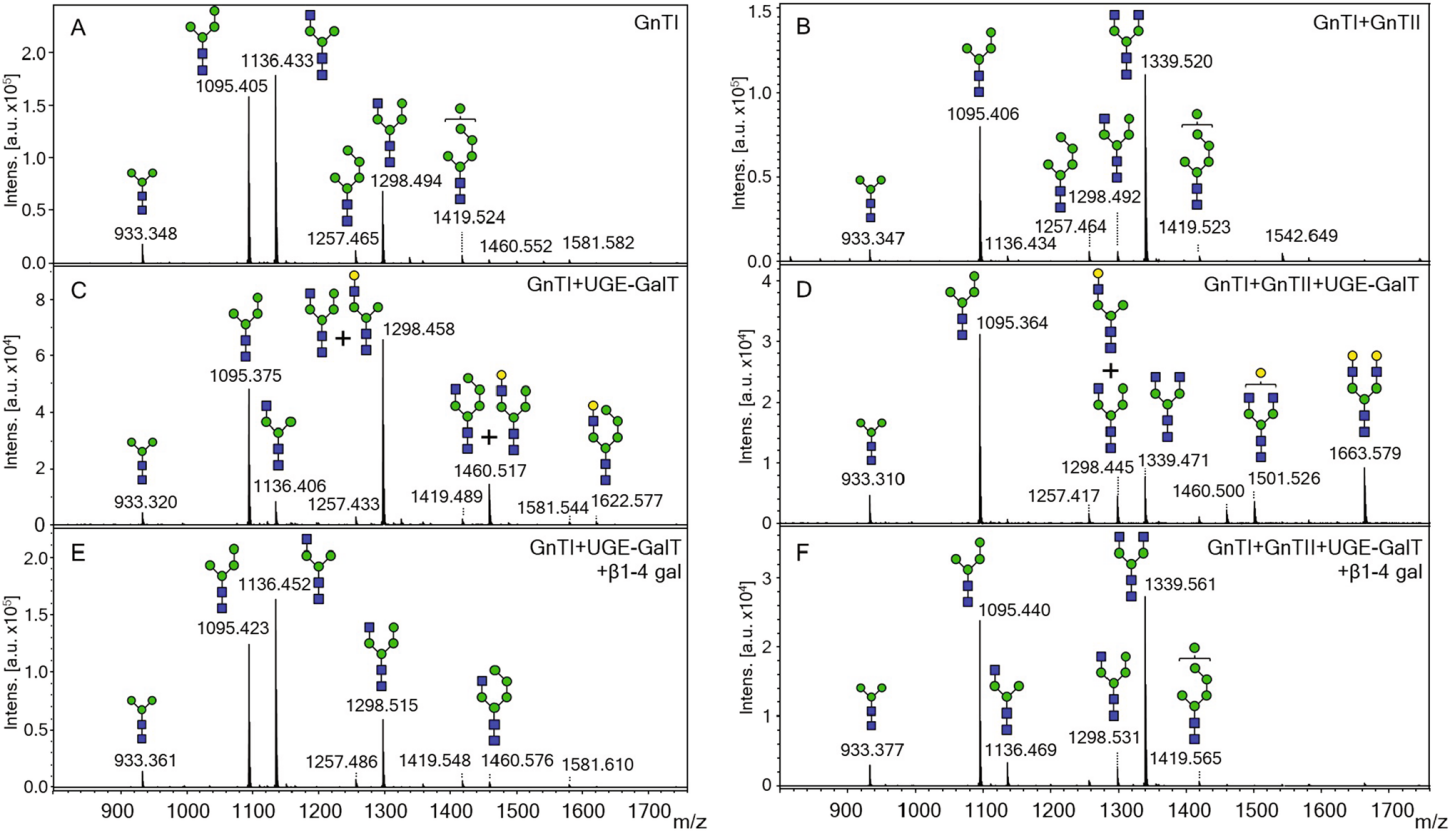
Representative MALDI-TOF MS spectra of N-glycans isolated from cell wall proteins in strain YMP17 expressing pAF22 (**A**), pAF21 (**B**), pAF22 and pSR02 (**C**), and pAF21 and pSR02 (**D**). MALDI-TOF MS spectra of **C** and **D** after β1-4 galactosidase digestion (**E** and **F**, respectively)

After demonstrating that the GalT and Uge1-GalT constructs had satisfactory in vivo activity towards hybrid-like glycans in *S. cerevisiae*, we next coexpressed them with GnTI, GnTII, and Yea4. As a result, mono- and digalactosylated complex-type glycans (Gal_1-2_GlcNAc_2_Man_3_GlcNAc_2_ or G1 and G2, respectively) were formed (Figs. 4 and 5). A higher relative abundance of G2 glycans was obtained with the Uge1-GalT construct compared to the construct without the epimerase (11.5% and 5.1%, respectively, Fig. 4, Table S3). However, the relative abundance of G2 was rather low with both constructs, and several incompletely processed as well as mannosylated structures were present. Sequential α1-2 mannosidase and β1-4 galactosidase digestion revealed that approximately 60% of all β1-2 linked GlcNAc residues were processed by GalT or Uge1-GalT, which was less than when expressing GalT or Uge1-GalT with GnTI and Yea4 only (Table 2). A vast majority of the signals at m/z 1298 and 1461 arose from galactosylated hybrid-like structures, as observed earlier. However, only 27% of the available G0 structures were converted to G2 glycans by the GalT construct (Table 2). When the Uge1-GalT construct was used instead, the conversion from G0 to G2 was higher (46%, Table 2). Thus, the UDP-glucose 4-epimerase domain did not seem to impact the overall efficiency of galactosylation but its presence increased the yield of G2 structures (Table S3).

Analysis of the efficiencies of the GnTI and GnTII reactions preceding the galactosylation step revealed that the GnTI efficiency decreased significantly when coexpressed with GnTII and GalT (Table 2), resulting in increased relative abundances of Man_3_GlcNAc_2_ and Man_4_GlcNAc_2_ structures (Fig. 4, Table S3). In addition, the GnTII efficiency decreased even more, as shown by the appearance of the hybrid-like GlcNAcMan_4_GlcNAc_2_ and GalGlcNAcMan_3-4_GlcNAc_2_ glycans. When the UDP-glucose 4-epimerase domain was included, both GnTI and GnTII processing efficiencies were somewhat increased (42% and 63%, respectively) but remained significantly lower than without GalT expression (Table 2). Therefore, the coexpression of GnTI, GnTII, GalT, and Yea4 seemed to have a negative impact on the activities of all three glycosyltransferases, and the presence of the Uge1p domain partially mitigated this effect. To confirm that GnTI and GnTII were expressed normally in the presence of the GalT or Uge1-GalT expression vector, we analyzed intracellular GnTI and GnTII levels by Western blot (Figure S2). Both GnTI and GnTII were detected albeit the apparent molecular weight of GnTII was slightly lower than expected, and their levels were not notably changed by GalT or Uge1-GalT expression. In order to better understand the physiological consequences of the overexpression of glycosyltranferases, we also measured the growth of the strains expressing GnTI only, GnTI and GnTII, and GnTI, GnTII, and UGE-GalT, respectively, to analyze whether the expression of multiple glycosyltransferases has a negative impact on viability. The expression of UGE-GalT did not hamper growth compared to the expression of GnTI or coexpression of GnTI and GnTII only; in fact, the growth rate was higher when UGE-GalT was expressed (Figure S3).

### Optimizing Gal_2_GlcNAc_2_Man_3_GlcNAc_2_ synthesis by growth medium supplementation

**Fig. 6:**
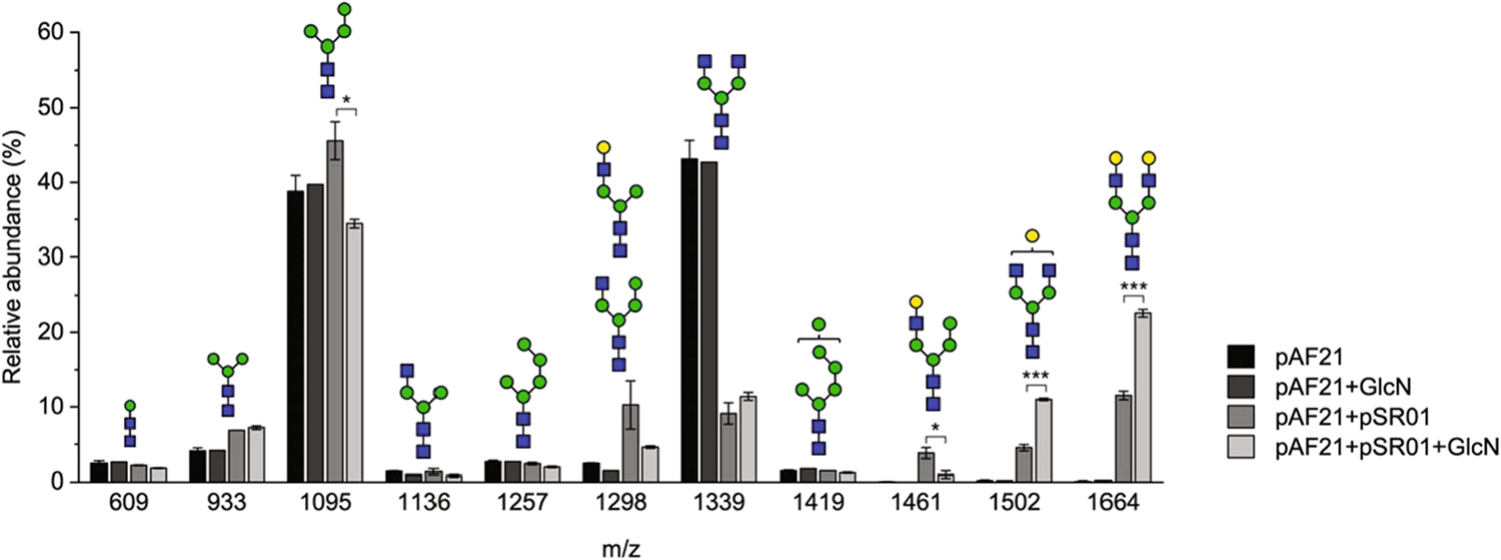
Relative abundances of N-glycans isolated from the cell walls of YMP17 expressing GnTI, GnTII, and *KlYEA4* (pAF21) or GnTI, GnTII, *KlYEA4*, and Uge1-GalT (pAF21 + pSR01) with and without glucosamine (GlcN) measured by MALDI-TOF MS. The data represent the mean ± SEM of three biological replicates, except for YMP17 expressing pAF21 with glucosamine the mean of two biological replicates is shown. *p* < 0.05 (*), *p* < 0.001 (***)

After defining the optimal genetic constructs, we tested whether the formation of G0 and G2 glycans could be further improved by growth medium optimization. Among several growth medium adjustments tested, we found that supplementation of 15 mM glucosamine doubled the relative abundance of formed G2 glycans in cell wall N-glycan samples (Fig. 6, Table S3). In these samples, the efficiencies of GnTI and GnTII reactions (53% and 88%, respectively) were significantly improved compared to samples grown without glucosamine and comparable to the efficiencies obtained without Uge1-GalT expression (Table 2). Thus, glucosamine supplementation seemed to compensate for the loss of GnTI and GnTII activities caused by GalT expression. However, supplementation of glucosamine had no impact on the glycan pattern when only GnTI, GnTII, and Yea4 were coexpressed.

**Fig. 7:**
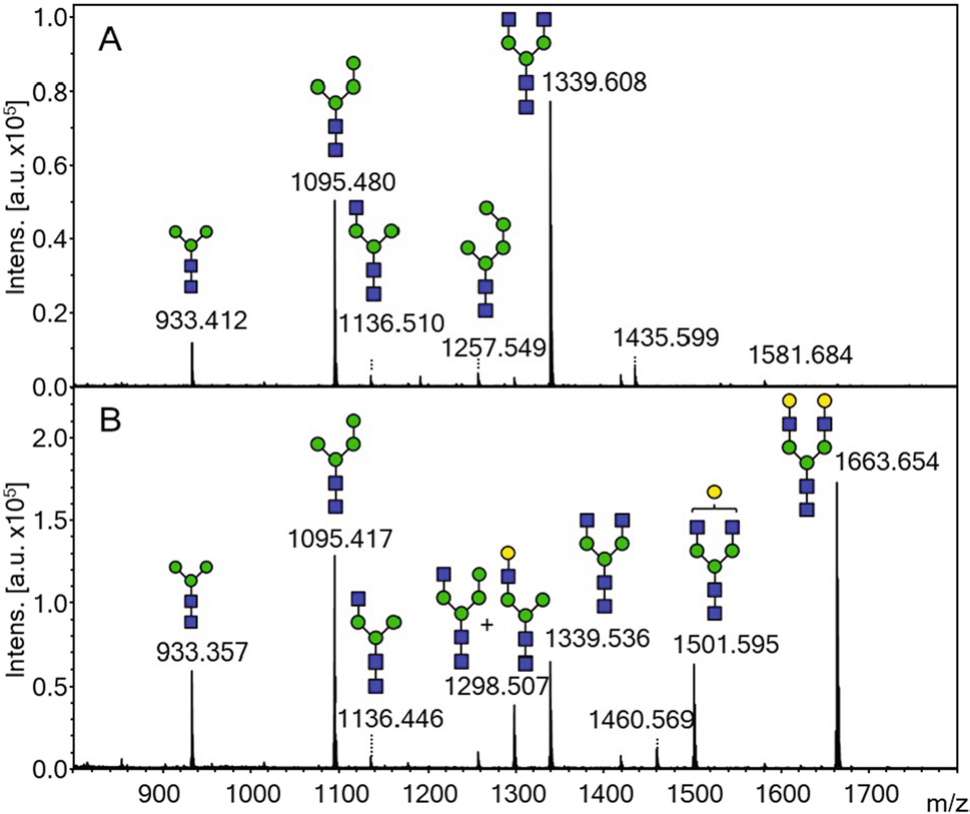
Representative MALDI-TOF MS spectra of N-glycans isolated from secreted proteins in strain YMP17 expressing GnTI, GnTII, and *KlYEA4* (**A**) and GnTI, GnTII, *KlYEA4*, and Uge1-GalT (**B**) grown in the presence of 15 mM glucosamine

We then utilized the optimized genetic constructs and growth medium to analyze the N-glycan pattern of the total secreted proteins, which is likely to provide a better representation of the glycan pattern in recombinantly produced proteins. As a result, we obtained nearly 50% relative abundance of the G0 structure when GnTI, GnTII, and Yea4 were expressed (Table S4). When coexpression of Uge1-GalT was included, G2 was the most prevalent glycan structure with up to 30% relative abundance (Fig. 7, Table S4). Compared to cell wall samples, N-glycans from secreted proteins generally contained higher amounts of the initial Man_3_GlcNAc_2_ glycans that were neither processed by GnTI nor mannosylated (Parsaie Nasab et al. 2013 and Tables S3 and S4). However, glucosamine supplementation restored the relative abundance of Man_3_GlcNAc_2_ in secreted proteins close to the levels found in cell wall samples.

Finally, to demonstrate the expression of a therapeutically relevant recombinant protein carrying galactosylated complex-type N-glycans, an IgG molecule was expressed in the strain with the optimized genetic constructs for GnTI, GnTII, and GalT activities and its N-glycans were analyzed. The N-glycan pattern of the expressed IgG contained galactosylated G1 and G2 structures along with G0 and other incompletely processed structures, although their relative abundances differed somewhat from the glycan patterns of cell wall and total secreted proteins (Figure S4).

### The interfering mannose is linked to the α1-6 branch of the trimannosyl core

Throughout our efforts to create GlcNAc-containing and galactosylated glycans, a significant amount of interfering mannosylation took place despite the optimization of G0-G2 biosynthesis pathway, as seen by the persistent presence of the Man_4_GlcNAc_2_ structure. The additional mannose can interfere with the GlcNAc transferase activities, and individual deletions of several known or putative α1-2 mannosyltransferases have failed to eliminate the mannosylation (Parsaie Nasab et al. 2013; Piirainen et al. 2016). Therefore, we aimed to gather more detailed information on the structure and formation of these interfering structures.

To confirm that no Man_4_GlcNAc_2_ is formed already during the LLO biosynthesis, we isolated LLOs from strain YMP17 and analyzed them with MALDI-TOF MS. LLOs of an Δ*alg3* Δ*alg11* yeast strain have been analyzed earlier by HPLC with [^3^H]mannose labeling (Parsaie Nasab et al. 2013), and the strain was reported to produce exclusively Man_3_GlcNAc_2_ LLOs. However, strain YMP17 contains several additional modifications compared to the strain analyzed earlier. Most importantly, the strain has been UV-mutagenized for improved growth. Additionally, as Man_3_GlcNAc_2_-PP-Dol is a poor substrate for the flippase and OST complex in the LLO biosynthesis pathway, the reduced glycosylation efficiency has been compensated by expression of an artificial flippase and the OST from *Leishmania brasiliensis* (Parsaie Nasab et al. 2013). Further on, the *MNN1* gene was also deleted to eliminate the formation of Man_5_GlcNAc_2_ and larger glycans (Piirainen et al. 2016). A MALDI-TOF MS spectrum of LLOs in YMP17 (Figure S5) shows the presence of Man_3_GlcNAc_2_ and a minor amount of Man_2_GlcNAc_2_, corresponding to the earlier results from the original Δ*alg3* Δ*alg11* strain (Parsaie Nasab et al. 2013) and confirming that Man_4_GlcNAc_2_ is only formed after the transfer of the glycan to a protein. N-glycan analysis of cell wall proteins in YMP17 in turn shows that Man_3_GlcNAc_2_ was almost entirely converted to Man_4_GlcNAc_2_ (Figure S5).

Two different isomeric structures can form Man_4_GlcNAc_2_ glycans, as the fourth mannose can be linked either to the Manα1-3 branch or to the Manα1-6 branch of the trimannosyl core. The formation of GlcNAcMan_4_GlcNAc_2_ upon expression of GnTI indicates that at least a part of the mannosyltransferase activity links the fourth mannose to the α1-6 linked branch, as the α1-3 linked branch has been processed by GnTI. However, it cannot be excluded that Man_4_GlcNAc_2_ is a mixture of both isomers. Especially mannosylation taking place in the α1-3 linked branch would result in interference with the GnTI reaction. Furthermore, it is not known whether a single or multiple mannosyltransferases contribute to the mannosylation. We performed high-energy CID MS/MS analysis of permethylated and underivatized Man_4_GlcNAc_2_ in positive and negative ion mode, respectively, to find out the position of the fourth mannose (Stephens et al. 2004; Domann et al. 2012). In-depth MS/MS analysis of Man_4_GlcNAc_2_ glycans from various strains by negative and positive CID revealed several diagnostic fragment ions indicating the presence of the additional mannose residue in the α1-6 arm of the trimannosyl core (Figure S6). Only weak or inconclusive fragmentation corresponding to the presence of the fourth mannose in the α1-3 arm was found. Thus, even though the presence of a second isomer could not be conclusively ruled out, the data suggest that at least a majority of Man_4_GlcNAc_2_ glycans contain the additional mannose in the α1-6 arm.

## Discussion

In this study, we conducted a comprehensive analysis on the factors affecting N-glycan processing and introduced a further step to the biosynthesis of human-compatible N-glycans in glycoengineered *S. cerevisiae*. Galactose residues serve important functions in N-glycans, such as enhancing the biological activity of antibodies (Reusch and Tejada 2015). Thus, we aimed to include the galactosylation step into an *S. cerevisiae* strain that can form complex-type N-glycans with terminal GlcNAc residues (Parsaie Nasab et al. 2013). We tested two alternative galactosyltransferase constructs: Golgi-targeted human GalT and a corresponding fusion protein developed by Bobrowicz et al. (2004) that additionally contains Uge1p from *S. pombe*, a yeast that natively produces galactose-containing glycans. Uge1p catalyzes the epimerization of UDP-glucose into UDP-galactose, which is used as a sugar donor for galactosyltransferases (Suzuki et al. 2010). In glycoengineered *P. pastoris*, expression of human GalT resulted in inefficient galactosylation, possibly due to a lack of UDP-galactose in the Golgi apparatus (Vervecken et al. 2004; Bobrowicz et al. 2004). Bobrowicz et al. reasoned that a Golgi-localized Uge1p could generate a local UDP-galactose supply for the GalT, and the Uge1-GalT fusion construct enabled efficient formation of Gal_2_GlcNAc_2_Man_3_GlcNAc_2_ glycans. This construct has also been used in other glycoengineered yeast strains (Jacobs et al. 2009; Wang et al. 2013).

In our experiments, both galactosyltransferase constructs provided efficient galactosylation of hybrid-like glycans. We used galactose as an inducer for the expression of the glycosyltransferases, which also provides an extracellular source of UDP-galactose precursor that can be imported into the cytosol and converted into UDP-galactose by Gal1p, Gal10p, and Gal7 (Lohr et al. 1995). It seems that in the presence of extracellular galactose, UDP-glucose 4-epimerase activity is not required for maintaining sufficient UDP-galactose levels in the Golgi apparatus. This is in agreement with the finding that UDP-galactose can be transported into the Golgi apparatus in *S. cerevisiae* although no UDP-galactose transporters have yet been identified (Roy et al. 1998). Also in *S. pombe*, *UGE1* was only required for cell surface galactosylation when grown in the absence of galactose (Suzuki et al. 2010). In contrast, *P*. *pastoris* cannot assimilate galactose due to the loss of galactose assimilating enzymes and therefore UDP-glucose 4-epimerase is critical for efficient galactosylation in this yeast.

Interestingly, the presence of the Uge1p domain increased the formation of complex-type G2 glycans even though the availability of UDP-galactose did not seem to be limited and the overall galactosylation efficiency was not strongly affected by Uge1p. This finding raises an interesting question about the role of the UDP-glucose 4-epimerase domain. The role of Golgi-localized Uge1p for the galactosylation reaction has also been questioned earlier, as NAD^+^ required for the catalytic activity of Uge1p is not known to be imported into the secretory pathway (De Pourcq et al. 2010). Also, our results suggest that rather than the epimerization reaction per se, the role of Uge1 in N-glycan galactosylation might be related to other aspects of the glycan maturation pathway, such as the activity or substrate specificity of GalT or the activity of GnTII.

The presence of the Uge1p domain increased the conversion from G0 to G2, which was relatively low compared to the extent of galactosylation in hybrid-like glycans. GalT does not process the acceptor sites at the α1-3 and α1-6 linked branches of the N-glycan with equal efficiencies (Pâquet et al. 1984; Blanken et al. 1984), and the presence of multiple substrate sites makes galactosylation of G0 kinetically a complex process (McDonald et al. 2018). Our data suggest that the inclusion of the Uge1p domain in the GalT construct possibly improved the efficiency of this process. The Uge1p domain can induce various structural changes to the GalT fusion protein potentially affecting the catalytic activity or branch specificity of GalT. These structural factors include the location of the catalytic domain relative to the membrane and the flexibility of the protein. GalT is also shown to exist in a dynamic equilibrium between monomeric and homodimeric states (Harrus et al. 2018), and this equilibrium could be affected by the Uge1p domain.

The Uge1p domain also mitigated the decrease in the GnTII activity that occurred upon GalT expression. The decreased activity of GnTII in the presence of GalT probably results from the competition of GalT and GnTII for the glycan substrates. GnTI, GnTII, and GalT need to process Man_3_GlcNAc_2_ in a sequential order to form G2 because GnTII requires the GlcNAcMan_3_GlcNAc_2_ structure created by GnTI as its substrate (Vella et al. 1984) and is unable to process galactosylated GalGlcNAcMan_3_GlcNAc_2_ structures (Bendiak and Schachter 1987; Kadirvelraj et al. 2018). However, GalT can also galactosylate hybrid-type glycans that have not been processed by GnTII. Localization of GalT too early in the secretory pathway relative to GnTII can cause premature galactosylation, preventing further glycan processing by GnTII. In our strains, GalT and GnTII were both expressed as fusions with Mnn2p targeting sequences. Despite some uncertainty regarding the localization of GnTII due to its unexpectedly low apparent molecular weight, the localization of GnTII and GalT activities overlapped as both complex-type Gal_1-2_GlcNAc_2_Man_3_GlcNAc_2_ and hybrid-like GalGlcNAcMan_3-4_GlcNAc_2_ glycans were formed, indicating that galactosylation took place both before and after the GnTII step. Mnn2p localization sequence was used for GnTII and Uge1-GalT also in *H. polymorpha*, where both hybrid and complex galactosylated glycans were formed similarly to our results (Wang et al. 2013). However, the level of premature galactosylation was very low in our strains when the optimal genetic constructs and growth medium were used. As the negative effect of GalT on GnTII efficiency was partially mitigated by the Uge1p domain, we could speculate that perhaps the inclusion of Uge1p domain in the GalT fusion protein might have an impact on either the activity of GnTII or the relative localization of GnTII and GalT, enabling a higher amount of GnTII processing prior to galactosylation.

Coexpression of GnTI, GnTII, Yea4, and GalT or Uge1-GalT decreased the efficiencies of all three glycosyltransferases compared to strains expressing only two of the glycosyltransferases. In addition to the factors mentioned above, the lowered efficiency of all three glycosyltransferases might also be caused by a limited capacity of a yeast cell to express heterologous enzymes. We expressed GnTI, GnTII, and GalT under the control of *GAL1* promoter that requires a transcriptional activator Gal4p for expression (Traven et al. 2006). When this promoter is used in multiple copies for heterologous gene expression, the amount of regulatory proteins can become limiting (Schultz et al. 1987), and as a consequence lower the expression levels of all three glycosyltransferases.

We also improved the efficiencies of GnTI and GnTII reactions, which create the substrate glycans for GalT, by changing their expression construct to a low copy number plasmid. In the cloning process, the *GAL10* promoter for GnTII was replaced by *GAL1* whereas the promoter for GnTI remained as *GAL1*. The lower copy number improved the GnTI activity, but it is not clear whether also the promoter change affected GnTII expression. Data regarding the relative strength of *GAL1* and *GAL10* promoters is not consistent, as *GAL10* promoter has resulted in lower (West et al. 1984; Yocum et al. 1984; Park et al. 2000), higher (Partow et al. 2010), or approximately equal expression levels compared to *GAL1* promoter (Cartwright et al. 1994; Elison et al. 2018). Expressing high levels of heterologous proteins and maintaining a high number of plasmid DNA can cause metabolic burden (Karim et al. 2013), affecting the general fitness of the cell and various cellular processes including glycan maturation. Lowering the copy number seemed to maintain sufficient intracellular glycosyltransferase levels while minimizing the cellular stress caused by their expression.

Expression of the *K. lactis* UDP-GlcNAc transporter improved the formation of G0 glycans as also reported earlier, suggesting that the supply of UDP-GlcNAc in the Golgi apparatus might be limited in yeast. Yeast cells can import extracellular glucosamine and convert it to UDP-GlcNAc, resulting in an elevated intracellular UDP-GlcNAc concentration (Bulik et al. 2003). We hypothesized that an additional increase in the intracellular UDP-GlcNAc concentration could further increase the formation of complex-type glycans. Interestingly, extracellular glucosamine had no impact in strains expressing GnTI, GnTII, and Yea4 but a positive impact was seen when the expression of Uge1-GalT was included. Without extracellular glucosamine, the GnTI and GnTII efficiencies were relatively low in this strain due to the negative effects of GalT on these glycosyltransferases, but glucosamine supplementation seemed to compensate for this activity loss. Taken together with the observation that the coexpression of Yea4 only increased the efficiency of GnTII, our results suggest that high GnTII activities can be obtained by improving the expression vector and the UDP-GlcNAc supply but an unidentified factor seems to limit the efficiency of GnTI to approximately 50%.

The limited GnTI activity could be connected to the Man_4_GlcNAc_2_ glycans that were formed in significant amounts in addition to the hybrid-like and complex glycans. Elimination of the additional α1-2 linked mannose residue has been attempted by deletion of various known or putative α1-2 mannosyltransferase genes including *MNN2*, *MNN5*, *KRE2*, *KTR1*, and *KTR3* (Parsaie Nasab et al. 2013) without success. We also tested deletions of *MNN9* and *VAN1*, members of the mannan polymerase complex I that elongates the α1-6 linked N-glycan outer chain and has been reported to also have α1-2 mannosyltransferase activity (Stolz and Munro 2002). However, no effects on glycan pattern were seen (unpublished data). It is possible that the mannosylation is either caused by an uncharacterized enzyme or multiple mannosyltransferases with overlapping activities. However, interfering α1-2 linked mannosylation has also been observed in other glycoengineered yeasts and eliminated or largely reduced by expression of GnTI and GnTII (Hamilton et al. 2003; Bobrowicz et al. 2004; Wang et al. 2013), suggesting that the mannosylation could also be prevented if sufficiently active and optimally localized GlcNAc transferases are used.

**Fig. 8:**
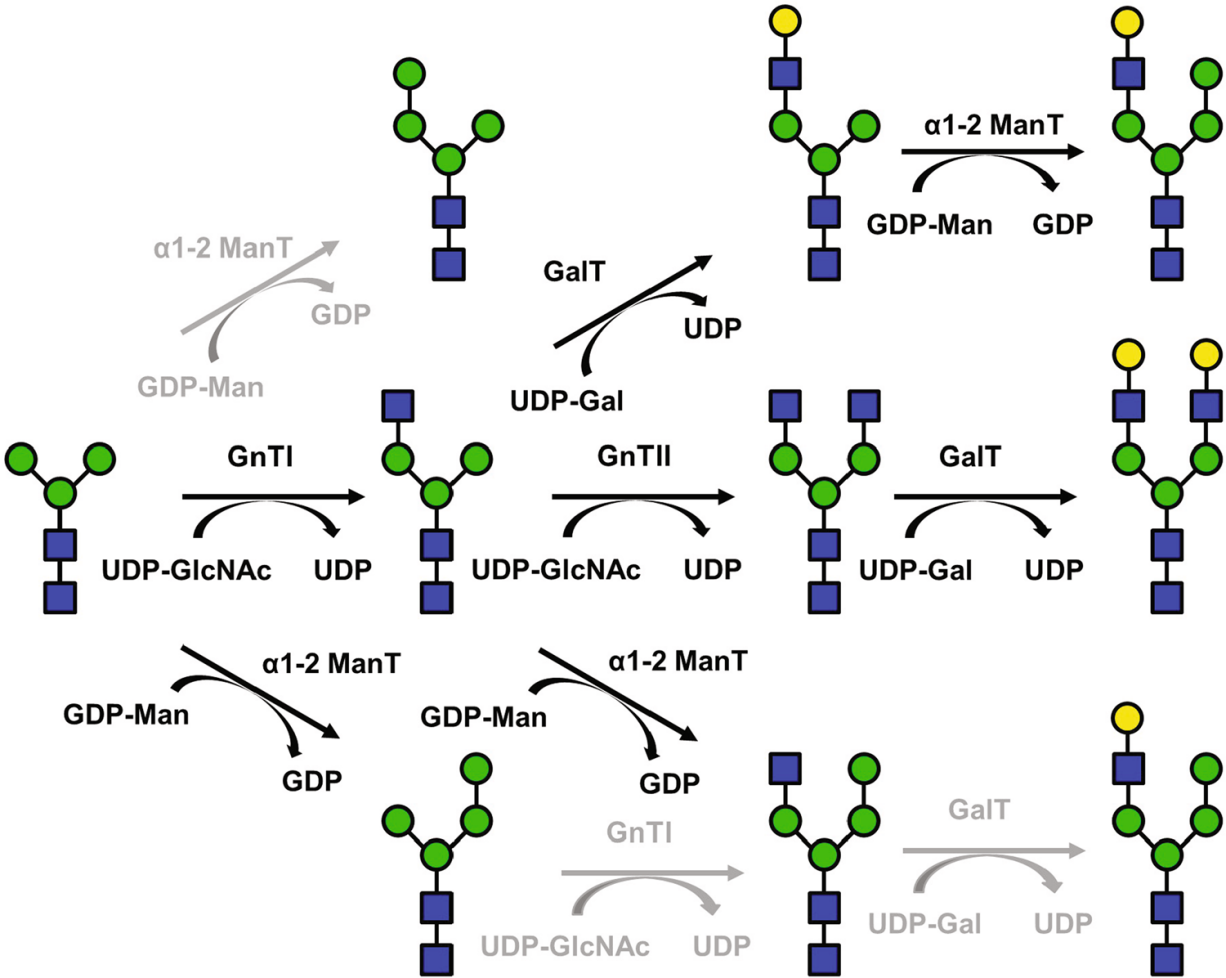
Biosynthetic pathway from trimannosyl core to galactosylated, complex-type N-glycans, and proposed side routes. Reactions unlikely to occur or occurring in only minor amounts are marked with grey arrows. α1-2 ManT, α1-2 mannosyltransferase

The position of the additional mannose in the trimannosyl core and the localization of the mannosyltransferase activity relative to GnTI and GnTII determines if screening of potential mannosyltransferase deletions or optimized expression and localization of GnTI or GnTII would more likely eliminate the interfering mannosylation. Our MS/MS data showed that the additional mannose residue was at least mostly linked to the α1-6 arm of the trimannosyl core. Thus, this mannose residue does not directly block the acceptor site for GnTI. Although the activity of GnTI towards Man_4_GlcNAc_2_ is not known, experimental data on various mammalian GnTIs suggests that GnTI can process glycans with varying α1-6 branch structures, including structures with glycosidic linkages at the C2 of the α1-6 mannose, albeit at somewhat lower activity than the native substrate glycan Man_5_GlcNAc_2_ (Oppenheimer et al. 1981; Oppenheimer and Hill 1981; Vella et al. 1984). Thus, if Man_4_GlcNAc_2_ glycans were formed in the glycan maturation process before the GnTI step, we assume that they would likely be processed by GnTI at least to some extent. However, very little GlcNAcMan_4_GlcNAc_2_ was formed when GnTI, GnTII, and Yea4 were coexpressed, suggesting that only Man_3_GlcNAc_2_ glycans are encountered by GnTI and the glycans not processed by GnTI would be converted to Man_4_GlcNAc_2_ later in the Golgi apparatus. Also our preliminary N-glycan data from whole cell extracts supports this presumption. Whole cell extracts that contain intracellular N-glycan intermediates in addition to readily processed cell wall N-glycans had a lower relative abundance of Man_4_GlcNAc_2_ compared to the cell wall samples, supporting the assumption that the α1-2-mannosyltransferase activity is not likely to occur very early in the secretory pathway (unpublished results). Thus, optimizing the catalytic activity of GnTI would be a primary target for further engineering. Based on the relative abundances of various glycans found in our samples, we constructed a route from Man_3_GlcNAc_2_ to G2 and interfering structures (Fig. 8).

The data in this work mostly represent the average glycan patterns found in cell wall and secreted proteins. While the cell wall protein fraction contains mostly GPI-anchored proteins, the secreted proteins represent a pool of soluble proteins. We expect that the observed glycan patterns provide realistic views on the glycan pattern to be expected for a recombinant protein. Indeed, our data on the glycan pattern of a purified recombinant antibody expressed in the yeast cells supports the notion that cell wall and secreted proteins are good indicators for the glycan pattern although a larger fraction of Man_3_GlcNAc_2_ remained unmodified. However, as glycoforms between different proteins and even between glycosylation sites within a protein can vary, the glycosylation pattern needs to be evaluated individually for each protein of interest when considering the potential applications of glycoengineered yeast in therapeutic protein production. Most therapeutic glycoproteins are a mixture of different glycoforms, and the range of required or acceptable glycoforms can vary depending on the product and the host cell line (Goh and Ng 2018). For example, the N-glycans of therapeutic antibodies are typically incompletely galactosylated and predominantly composed of fucosylated and nonfucosylated G0, G1, and G2 structures (Reusch and Tejada 2015). Thus, instead of homogeneous production of single glycoforms, the ability to produce glycan patterns comparable to current production platforms using yeast could be more advantageous. In addition to optimizing the glycosylation pattern, increasing the production levels of foreign proteins in yeast is essential in the context of therapeutic protein production, and our research group has also accomplished improvements in this area (Koskela et al. 2020).

## Supplementary Information

Below is the link to the electronic supplementary material.

Supplementary file1 (PDF 628 KB)

## Data Availability

All materials are shared with academic institutions upon request and execution of a material transfer agreement.
