# Supplementary material for: Production of galactosylated complex-type N-glycans in glycoengineered Saccharomyces cerevisiae
Source: Appl Microbiol Biotechnol. 2021 Dec 15;106(1):301–15. doi: 10.1007/s00253-021-11727-8 (PMC8720083; doi:10.1007/s00253-021-11727-8)
Supplement: Supplementary file 1 — Supplementary file1 (PDF 628 KB) [file 253_2021_11727_MOESM1_ESM.pdf]

Supporting information for:

Applied Microbiology and Biotechnology

Production of galactosylated complex type glycans in glycoengineered *Saccharomyces cerevisiae*

Mari A Piirainen, Heidi Salminen and Alexander D. Frey*

Aalto University, Department of Bioproducts and Biosystems, Espoo, Finland

*Corresponding author:

Alexander Frey,

alexander.frey@aalto.fi

phone: +358 50 411 65 06

fax: +358 9 462373

Supplementary methods

Western blotting

Cell pellet corresponding to 10 OD_{600} units was resuspended in 200 µl of SDS sample buffer (25 mM Tris base, 192 mM glycine, 0.1% SDS) with 50 mM DTT and 2 µl of Protease Inhibitor Cocktail (Sigma P8215) and lysed with 200 µl of 0.5 mm glass beads in a cell disruptor at +4°C for 10 min. Samples were centrifuged at 21 000 g, +4°C for 5 min to remove cell debris and unbroken cells. The supernatant was adjusted to SDS-PAGE sample buffer (62.5 mM Tris-HCl pH 6.8, 2 % SDS, 10 % glycerol, 50 mM DTT and 0.005 % bromophenol blue) and 20 µl samples were run on a 7.5 % SDS-PAGE gel and transferred onto a nitrocellulose membrane. GnTI and GnTII were detected with 1:12 000 dilution of anti-FLAG® M2 antibody produced in mouse (Sigma) and 1:80 000 dilution of peroxidase-conjugated anti-mouse IgG antibody (Sigma). GalT was detected with 1:2400 dilution of polyclonal human B4GALT1 antibody produced in rabbit (Thermo Fisher Scientific, Waltham, MA, USA) and 1:50 000 dilution of peroxidase-conjugated anti-rabbit IgG antibody (Sigma). Signals were detected with Supersignal™ West Pico Chemiluminescent Substrate (Thermo Fisher Scientific, Waltham, MA, USA).

Isolation of LLOs

YMP17 was grown for LLO isolation in complete SD medium, and cells were collected at mid-log phase. LLOs were isolated as described by (Zufferey et al. 1995) with some modifications. In short, 50 OD_{600} units of cells were lysed by vortexing with glass beads for 5 minutes, and vortexing was repeated after adding 50 µl of DM (dichloromethane:methanol, 3:2). The cell suspension was washed four times with 4 ml of DM, vortexed for 5 min and centrifuged (5 min 4500 *g*). Washing was repeated four times with 4 ml of UP (dichloromethane:methanol:water, 3:48:47) containing 4 mM $MgCl_2$, and two times with 4 ml of UP. LLOs were repeatedly extracted with 4 ml and two times with 3 ml of DMW (dichloromethane:methanol:water, 10:10:3) and dried under nitrogen gas at 37°C. For release of oligosaccharides from lipids, LLOs were dissolved in 35 µl isopropanol and hydrolyzed by incubating for 45 minutes at 95 °C with 1 ml of 20 mM HCl. Released oligosaccharides were extracted with 5 ml of DM, and extraction was repeated by addition of 3 ml of DM and 400 µl of water. Samples were dried under nitrogen gas at 60 °C and dissolved in 80 µl of water. Prior to MS analysis, LLO samples were purified with HyperSep Hypercarb SPE tips (Thermo Scientific, USA) and concentrated by evaporation.

Generation of antibody expression strain

The expression cassettes for IgG were introduced into strain YMP17 by integrating a DraIII-linearized plasmid pEK12, containing the ORFs for the heavy and light chains of anti-CD20 IgG (de Ruijter et al. 2016), into the *HIS3* locus of YMP17.The resulting strain YMP18 was transformed with plasmids pAF21 and pSR01.

Antibody expression and purification

Cultivations for antibody expression were done as described for the production and analysis of secreted proteins in a medium supplemented with 15 mM glucosamine, 20 mM phosphate buffer, pH 6.5 and 0.1 μg/ml BSA. The antibody was purified from four replicate cultures after pooling the cleared and filtrated culture supernatant. The supernatant was adjusted to 100 mM NaCl, 20 mM Tris, pH 7.2. A one ml protein G sepharose column was pre-equilibrated with 100 mM NaCl, 20 mM Tris, pH 7.2 and loaded at a flow rate of 100 ml / hour. The column was washed with 10 column volumes of 100 mM NaCl, 20 mM Tris, pH 7.2 and the bound antibody was eluted with 5 ml 1 M glycine, pH 3.0. The eluate was neutralized using 1 M Tris, pH 9.0. The eluate was concentrated and washed with water to a volume of 200-300 μl using centrifugal concentrators with a molecular weight cut-off of 10 000 Da (Sartorius, Göttingen, Germany). N-glycans were released from the antibody and prepared for MALDI-TOF MS analysis as described for cell wall and secreted proteins.

Small scale cultivations

Small scale cultivations were conducted with YMP17 strains transformed with plasmid pairs pEK17 and pEK7, pAF22 and pEK7, pAF21 and pEK7 and pAF21, respectively. Colonies were inoculated into 3 ml of SD medium lacking uracil and leucine, 0.2 M sorbitol and 2% raffinose and grown at 28 ºC, 250 rpm for 48 hours. Cultures were diluted into 3 ml of fresh medium to an OD_{600} of 0.2 and allowed to grow at 28 ºC, 250 rpm for 20 to 24 hours.

Cultures were diluted in one ml of fresh medium to an OD_{600} of 0.1 and 100 μl of this cell suspension was dispensed into round-bottom 96-well plates. Induced (2% galactose) and non-induced cells suspensions were prepared. The 96-well plate cultivations were conducted in a Cytation 3 multi-mode reader (Bio-Tek, Winooski, VT, USA) at 28 ºC and using a linear shaking mode. OD_{600} was measured every 15 minutes for 63 hours. OD_{600} readings of technical plate replicates were averaged, and growth curves represent means and standard errors from 5 to 7 independent colonies grown in two separate rounds of cultivations.

Supplementary figures

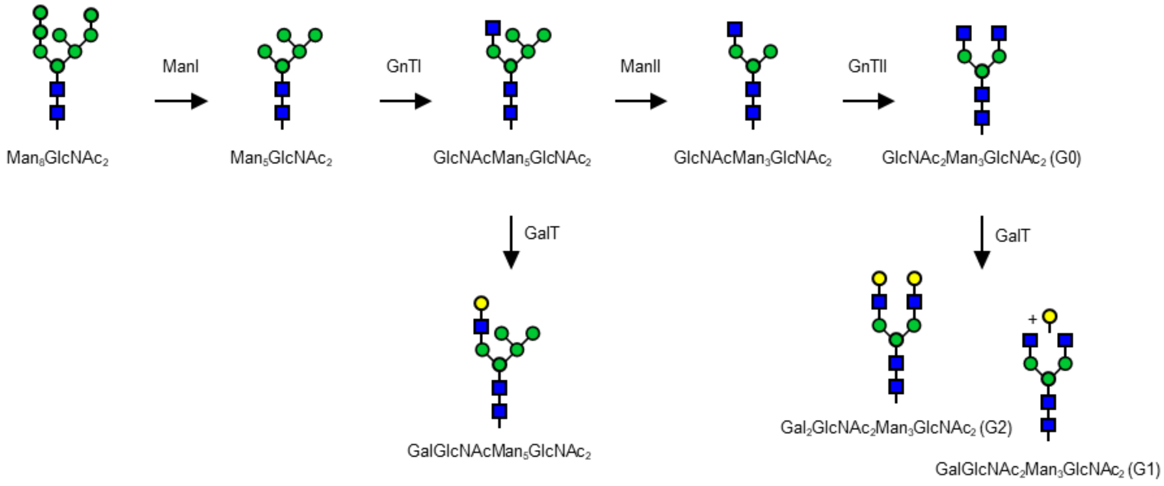

Figure S1. Schematic presentation of the processing of N-glycans in mammalian Golgi apparatus. ManI, Golgi α-mannosidase IA/IB/IC; ManII, Golgi α-mannosidase II.

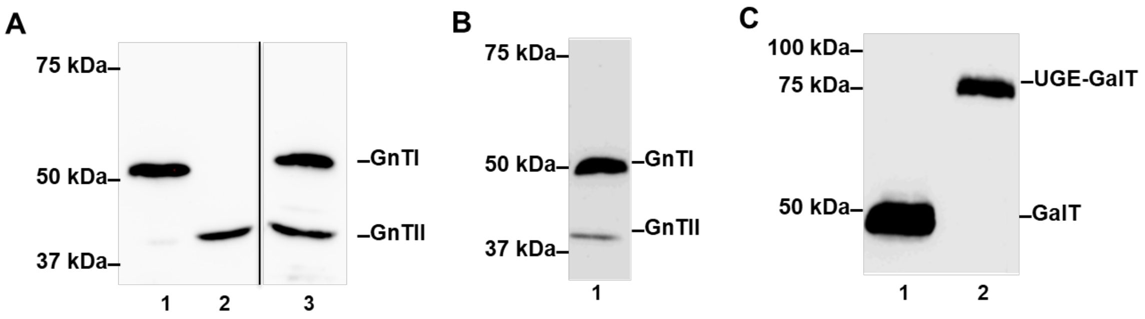

Figure S2. Western blot analysis of GnTI, GnTII, GalT and UGE-GalT fusion proteins. GnTI (51.5 kDa) and GnTII (47.2 kDa) were analyzed from cell extracts of the wild type yeast strain SS328 expressing GnTI (lane 1), GnTII (lane 2) and both GnTI and GnTII (lane 3) to confirm their identities (A). GnTI (51.5 kDa) and GnTII (47.2 kDa) were analyzed from cell extracts of YMP17 expressing GnTI and GnTII with GalT in plasmids pSKH01 and pSR02 (B). GalT (43.4 kDa) and UGE-GalT (83.0 kDa) were analyzed from cell extracts of YMP17 expressing GnTI and GnTII with GalT (lane 1) or UGE-GalT (lane 2) in plasmids pSKH01 and pSR02 or pSR01, respectively (C).

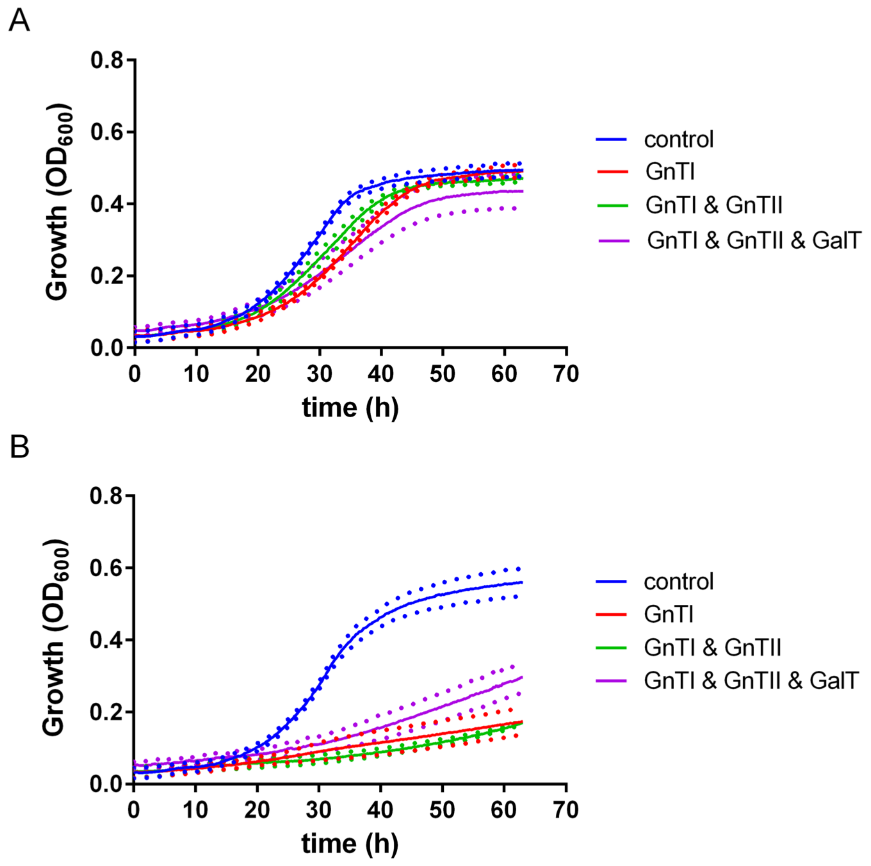

Figure S3. Small scale cultivations were conducted with YMP17 strains transformed with plasmid pairs pEK17 and pEK7 (control), pAF22 and pEK7 (GnTI), pAF21 and pEK7 (GnTII & GnTII) and pAF21 and pRS01 (GnTII & GnTII& GalT), respectively. Cultures were grown in the absence (A) and presence (B) of 2% galactose. The data represent mean (lines) and standard error (dotted lines) of 5 to 7 independent colonies.

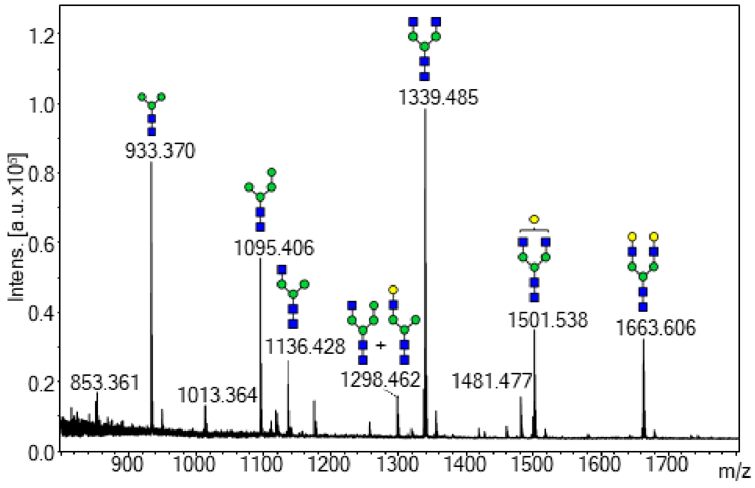

Figure S4. MALDI-TOF MS spectrum of N-glycans isolated from IgG produced in strain YMP18 harboring plasmids pAF21 and pSR01.

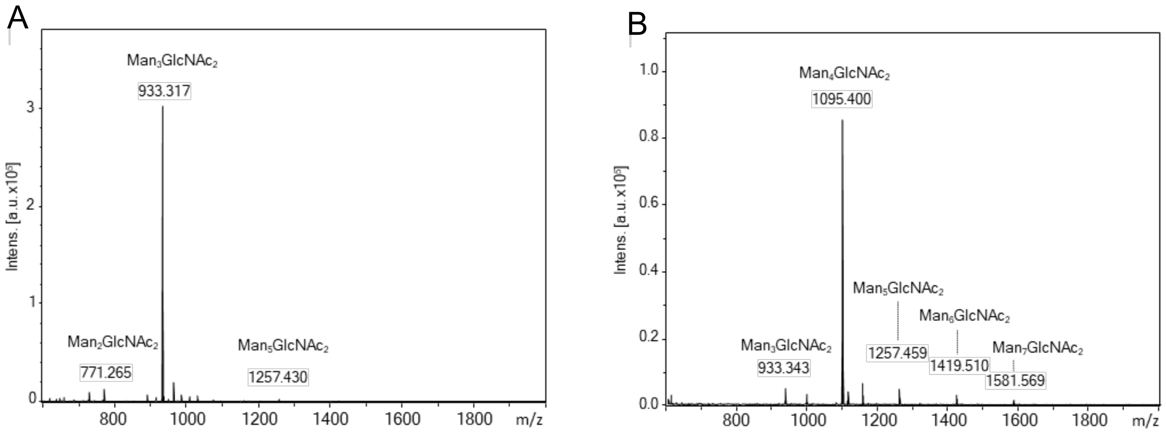

Figure S5. MALDI-TOF MS spectra of LLOs (A) and cell wall N-glycans (B) isolated from strain YMP17 grown in SD medium.

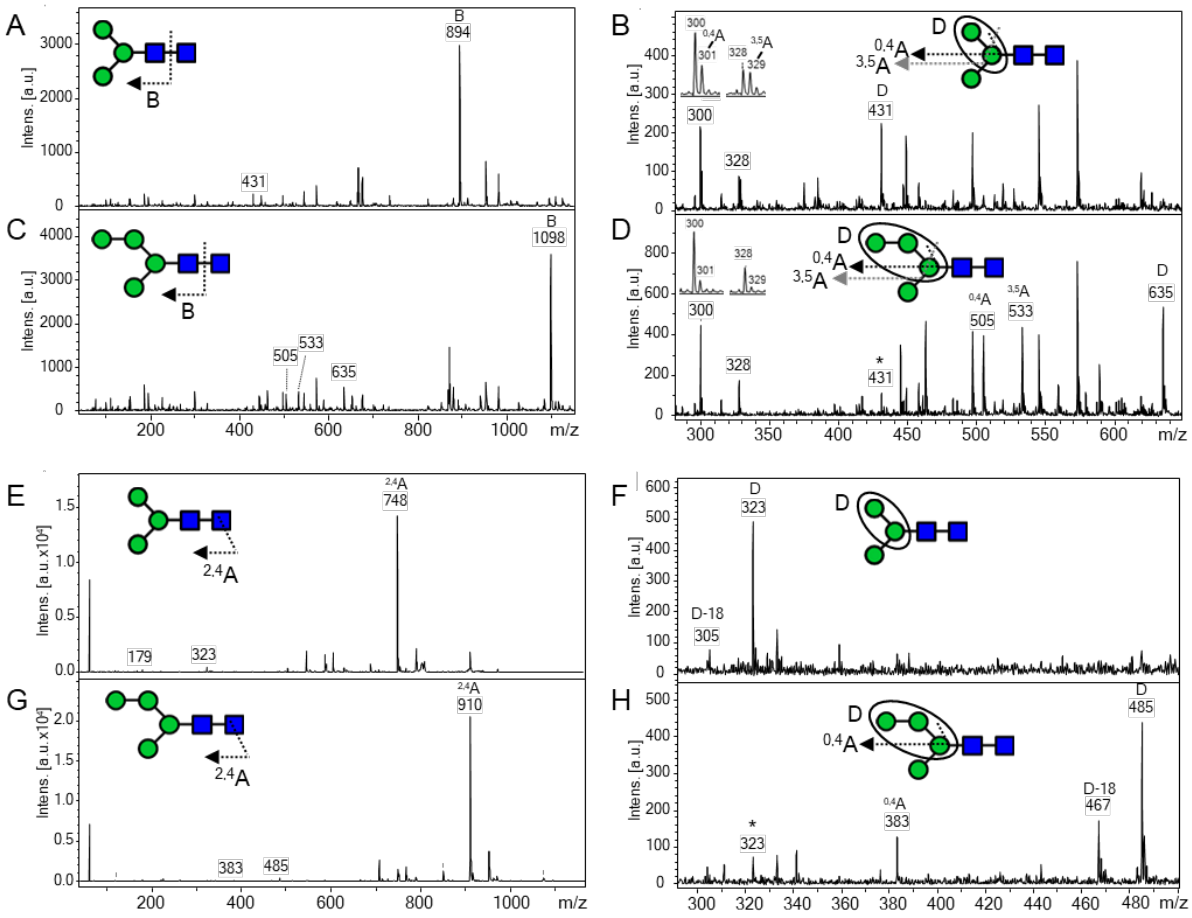

Figure S6. MALDI-TOF CID MS/MS analysis of permethylated and nonderivatized $Man_3GlcNAc_2$ and $Man_4GlcNAc_2$ N-glycans isolated from whole cell samples of YMP17 (A-D) and YMP17 expressing GnTI and GnTII in plasmid pSKH01 (E-H). Whole cell samples were prepared by TCA precipitation as described earlier (Piirainen and Frey 2020). Permethylated samples (A-D) were measured in positive ion mode and nonderivatized samples (E-H) in negative ion mode. MS/MS spectrum of permethylated $Man_3GlcNAc_2$ (m/z 1171, A) and the m/z range containing ^{3,5}A, ^{0,4}A and D fragments characteristic for an unoccupied α1-6 arm of Manα1-3(Manα1-6)Man-R according to Stephens et al. (2004) (B). MS/MS spectrum of permethylated $Man_4GlcNAc_2$ (m/z 1375, C), and the m/z range containing ^{3,5}A, ^{0,4}A and D fragments specific for the α1-6 arm of Manα1-3(Manα1-6)Man-R containing an additional mannose (D). The weak signals at m/z 301 and 329 in permethylated $Man_4GlcNAc_2$ (inlay of D) were considered as isotopic peaks of signals at m/z 300 and 328, respectively, rather than ^{3,5}A and ^{0,4}A fragments based on the intensity ratios and their presence also in hybrid-like $GlcNAcMan_4GlcNAc_2$ glycan (unpublished data). Negative MS/MS spectra of $Man_3GlcNAc_2$ (m/z 972, E) and the m/z range containing the D and D-18 fragments characteristic for an unoccupied α1-6 arm of Manα1-3(Manα1-6)Man-R according

to Domann et al. (2012) (F). Negative MS/MS spectra of $Man_4GlcNAc_2$ (m/z 1134, G), and the m/z range containing the D, D-18 and ^{0,4}A fragments specific for the α1-6 arm of Manα1-3(Manα1-6)Man-R containing an additional mannose (H). The low-intensity signals at m/z 323 and 431 indicated with an asterisk potentially result from an unidentified fragment or a contamination rather than an unoccupied α-1,6 arm based on their presence also in hybrid-like $Gal_{0-1}GlcNAcMan_4GlcNAc_2$ glycans (unpublished data).

Supplementary tables

Table S1. List of oligonucleotides used in this work.

Name	Sequence	Target
OMP88	AACAAATACATGGATGAGAACACGTCGATGACAGGAGTTC	SpUge1p-GSGG-GalT ORF
OMP89	AACAAATACATGGATGAGAACACGTCGGGTAGGGATTTGTCAAGATTACC	GalT ORF
OMP90	TGACATAACTAATTACATGACTCGAGCTATGATGGAGTTCC	SpUge1p-GSGG-GalT and GalT ORFs
OMP97	AAACCCCGGATTCTAGAACTAGTATGCTGCTTACCAAAAGG	Mnn2p-GnTII-FLAG ORF
OMP98	ACATAACTAATTACATGACTCGAGGTCGACGGTATCGATAAGC	Mnn2p-GnTII-FLAG ORF
OMP104	CTTCCTTTCTCGCCACGTTCGCCGGCTAGTACGGATTAGAAGCC	Mnn2p-GnTII-FLAG expression cassette
OMP105	TTTAGAGCTTGACGGGGAAAGCCGGCGGCCGCAAATTAAAGCCT	Mnn2p-GnTII-FLAG expression cassette
OMP108	ACTAAAGGGAACAAAAGCTGGAGGATATCGAGCTCTAGTACGGATTAGAAG	Kre2p-GnTI-FLAG expression cassette
OMP109	GCGGCTTCTAATCCGTACTAGAGGATATCTGCTGCAAGGCGATTAAG	Kre2p-GnTI-FLAG expression cassette

Table S2. Relative abundances of the N-glycan structures detected in the MALDI-TOF MS spectra obtained from the cell wall N-glycans of strain YMP17 expressing GnTI. Structures with a relative abundance of at least 1% are shown.

**Glycan structure**	**GnTI**	**GnTI+Yea4**	**GnTI+Yea4 +GalT**	**GnTI+Yea4 +Uge1-GalT**
$Man_1GlcNAc_2$	1.9 %	2.2 %	1.9 %	1.9 %
$Man_3GlcNAc_2$	4.4 %	4.7 %	5.5 %	5.0 %
$Man_4GlcNAc_2$	36.4 %	39.0 %	38.3 %	33.5 %
$GlcNAcMan_3GlcNAc_2$	30.4 %	34.8 %	8.5 %	7.9 %
$Man_5GlcNAc_2$	2.4 %	2.4 %	2.0 %	1.8 %
$GlcNAcMan_4GlcNAc_2$ or $GalGlcNAcMan_3GlcNAc_2$	19.6 %	12.0 %	34.0 %	39.3 %
$Man_5GlcNAc_2$	1.4 %	1.4 %	1.3 %	1.2 %
GlcNAcMan5$GlcNAc_2$ or $GalGlcNAcMan_4GlcNAc_2$	0.6 %	0.6 %	6.8 %	7.7 %

Table S3. Relative abundances of the N-glycan structures detected in the MALDI-TOF MS spectra obtained from the cell wall N-glycans of strain YMP17 expressing GnTI and GnTII. Structures with a relative abundance of at least 1% are shown. GlcN, glucosamine

**Glycan structure**	**GnTI+GnTII (high copy)**	**GnTI+GnTII**	**GnTI+GnTII +Yea4**	**GnTI+GnTII +Yea4 +GlcN**	**GnTI+GnTII +Yea4+GalT**	**GnTI+GnTII +Yea4 +Uge1-GalT**	**GnTI+GnTII +Yea4+Uge1-GalT +GlcN**
$Man_1GlcNAc_2$	1.4 %	2.0 %	2.5 %	2.6 %	2.6 %	2.2 %	1.8 %
$Man_3GlcNAc_2$	5.3 %	5.5 %	4.1 %	4.1 %	8.2 %	6.9 %	7.2 %
$Man_4GlcNAc_2$	53.4 %	40.8 %	38.8 %	39.7 %	48.0 %	45.6 %	34.5 %
$GlcNAcMan_3GlcNAc_2$	3.6 %	4.2 %	1.4 %	1.0 %	0.8 %	1.3 %	0.8 %
$Man_5GlcNAc_2$	4.6 %	2.8 %	2.7 %	2.7 %	2.4 %	2.4 %	1.9 %
$GlcNAcMan_4GlcNAc_2$ or $GalGlcNAcMan_3GlcNAc_2$	5.9 %	6.2 %	2.4 %	1.5 %	12.8 %	10.3 %	4.6 %
$GlcNAc_2Man_3GlcNAc_2$ (GO)	21.1 %	31.9 %	43.1 %	42.7 %	9.8 %	9.1 %	11.4 %
$Man_5GlcNAc_2$	2.3 %	2.0 %	1.5 %	1.7 %	1.5 %	1.4 %	1.2 %
$GlcNAcMan5GlcNAc_2$ or $GalGlcNAcMan_4GlcNAc_2$	0.2 %	0.1 %	0.0 %	0.0 %	4.1 %	3.8 %	0.9 %
$GalGlcNAc_2Man_3GlcNAc_2$ (G1)	0.3 %	0.2 %	0.2 %	0.2 %	3.5 %	4.5 %	11.0 %
$GlcNAc_3Man_3GlcNAc_2$	0.8 %	2.2 %	1.6 %	1.6 %	0.3 %	0.1 %	0.3 %
$Gal_2GlcNAc_2Man_3GlcNAc_2$ (G2)	0.0 %	0.2 %	0.1 %	0.3 %	5.1 %	11.5 %	22.6 %

Table S4. Relative abundances of the N-glycan structures detected in the MALDI-TOF MS spectra obtained from N-glycans of secreted proteins in strain YMP17 expressing GnTI and GnTII. Structures with a relative abundance of at least 1% are shown. GlcN, glucosamine.

**Glycan structure**	**GnTI+GnTII +Yea4**	**GnTI+GnTII +Yea4+GlcN**	**GnTI+GnTII +Yea4 +Uge1-GalT**	**GnTI+GnTII +Yea4+Uge1 -GalT+GlcN**
$Man_1GlcNAc_2$	1.5 %	1.8 %	1.2 %	1.0 %
$Man_3GlcNAc_2$	10.6 %	7.4 %	16.8 %	10.0 %
$Man_4GlcNAc_2$	37.5 %	33.6 %	33.7 %	23.2 %
$GlcNAcMan_3GlcNAc_2$	2.3 %	2.0 %	2.0 %	1.3 %
$Man_5GlcNAc_2$	2.7 %	2.4 %	2.4 %	1.8 %
$GlcNAcMan_4GlcNAc_2$ or $GalGlcNAcMan_3GlcNAc_2$	2.5 %	1.7 %	13.0 %	6.4 %
$GlcNAc_2Man_3GlcNAc_2$ (G0)	40.0 %	47.8 %	7.2 %	11.9 %
$Man_5GlcNAc_2$	2.0 %	2.0 %	1.7 %	1.3 %
GlcNAcMan5GlcNAc$_2$ or $GalGlcNAcMan_4GlcNAc_2$	0.0 %	0.0 %	3.8 %	2.1 %
$GalGlcNAc_2Man_3GlcNAc_2$ (G1)	0.0 %	0.0 %	3.6 %	11.2 %
$Gal_2GlcNAc_2Man_3GlcNAc_2$ (G2)	0.0 %	0.1 %	14.3 %	28.1 %

References

de Ruijter JC, Koskela E V., Frey AD (2016) Enhancing antibody folding and secretion by tailoring the *Saccharomyces cerevisiae* endoplasmic reticulum. Microb Cell Fact 15:87. https://doi.org/10.1186/s12934-016-0488-5

Domann P, Spencer DIR, Harvey DJ (2012) Production and fragmentation of negative ions from neutral N-linked carbohydrates ionized by matrix-assisted laser desorption/ionization. Rapid Commun Mass Spectrom 26:469–479. https://doi.org/10.1002/rcm.5322

Piirainen MA, Frey AD (2020) Investigating the role of ERAD on antibody processing in glycoengineered Saccharomyces cerevisiae. FEMS Yeast Res 20. https://doi.org/10.1093/femsyr/foaa002

Stephens E, Maslen SL, Green LG, Williams DH (2004) Fragmentation characteristics of neutral n-linked glycans using a MALDI-TOF/TOF tandem mass spectrometer. Anal Chem 76:2343–2354. https://doi.org/10.1021/ac030333p

Zufferey R, Knauer R, Burda P, Stagljar I, te Heesen S, Lehle L, Aebi M (1995) STT3, a highly conserved protein required for yeast oligosaccharyl transferase activity in vivo. EMBO J 14:4949–60
